# Lewis Acid-Catalyzed Carbonyl-Ene Reaction: Interplay between Aromaticity, Synchronicity, and Pauli Repulsion

**DOI:** 10.1021/acs.joc.3c01059

**Published:** 2023-07-24

**Authors:** Humberto A. Rodríguez, Daniel A. Cruz, Juan I. Padrón, Israel Fernández

**Affiliations:** † Instituto de Productos Naturales y Agrobiología, Consejo Superior de Investigaciones Científicas (IPNA-CSIC), Avda. Astrofísico Francisco Sánchez 3, 38206 La Laguna, Tenerife, Islas Canarias, Spain; ‡ Departamento de Química Orgánica I and Centro de Innovación en Química Avanzada (ORFEO-CINQA), Facultad de Ciencias Químicas, Universidad Complutense de Madrid, 28040 Madrid, Spain

## Abstract

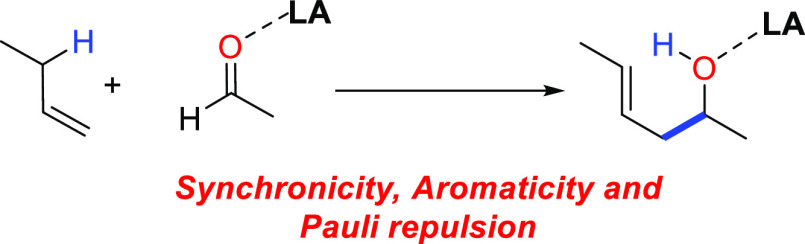

The physical factors governing the catalysis in Lewis acid-promoted carbonyl-ene reactions have been explored in detail quantum chemically. It is found that the binding of a Lewis acid to the carbonyl group directly involved in the transformation greatly accelerates the reaction by decreasing the corresponding activation barrier up to 25 kcal/mol. The Lewis acid makes the process much more asynchronous and the corresponding transition state less in-plane aromatic. The remarkable acceleration induced by the catalyst is ascribed, by means of the activation strain model and the energy decomposition analysis methods, mainly to a significant reduction of the Pauli repulsion between the key occupied π-molecular orbitals of the reactants and not to the widely accepted stabilization of the LUMO of the enophile.

## Introduction

The Alder-ene reaction, named after its discoverer Alder in 1943,^1^ constitutes one of the most fundamental reactions in organic chemistry. This transformation belongs to the family of group transfer pericyclic reactions and forms a C–C bond with concomitant 1,5-hydrogen shift by the reaction of an alkene bearing an allylic hydrogen atom (ene) with a multiple bond (enophile).^2^ Owing to its compatibility with a number of functional groups attached either to the ene or enophile moieties, this process has been widely applied to the synthesis of complex molecules including natural products.^3^ A particular type of Alder-ene reaction is that involving a carbonyl group as an enophile, known as the carbonyl-ene reaction.^4^ This process has been also widely applied to the formation of homoallylic alcohols and, similarly, has been used in the synthesis of complex molecules such as, for instance, (+)-steenkrotin A^5^ or (±)-andrastin C,^6^ among others.

**Scheme 1:**
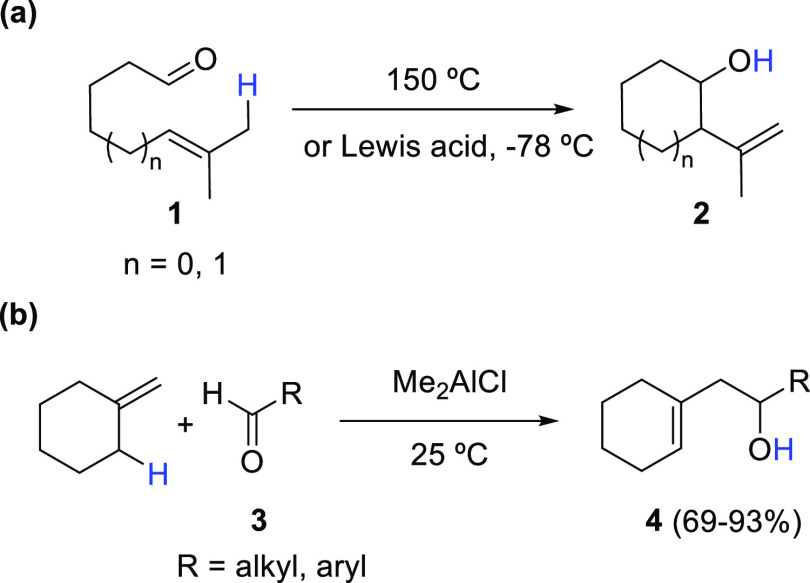
Representative Examples of Lewis Acid-Catalyzed Carbonyl-Ene Reactions

Similar to the parent ene reaction involving alkenes as enophiles, the analogous carbonyl-ene reactions also exhibit relatively high barriers, which is translated into high reaction temperatures (in the range of 150–300 °C) limiting in many instances the scope of the transformation. Despite this, the carbonyl moiety can be activated by complexation with a Lewis acid leading to a significant acceleration of the transformation, which makes these reactions feasible even at room temperature or below.^7^ For instance, whereas the intramolecular carbonyl-ene reactions involving the unactivated unsaturated aldehydes **1** produce alcohols **2** upon heating at 150 °C, the analogous process under Lewis acid catalysis produces the same reaction product in good yields at −78 °C (Scheme 1a).^8^ Similarly, good to excellent reaction yields of unsaturated alcohols **4** are achieved in the room temperature Me_2_AlCl-catalyzed intermolecular reaction involving aldehydes **3** and methylenecyclohexane (Scheme 1b).^9^

**Scheme 2:**
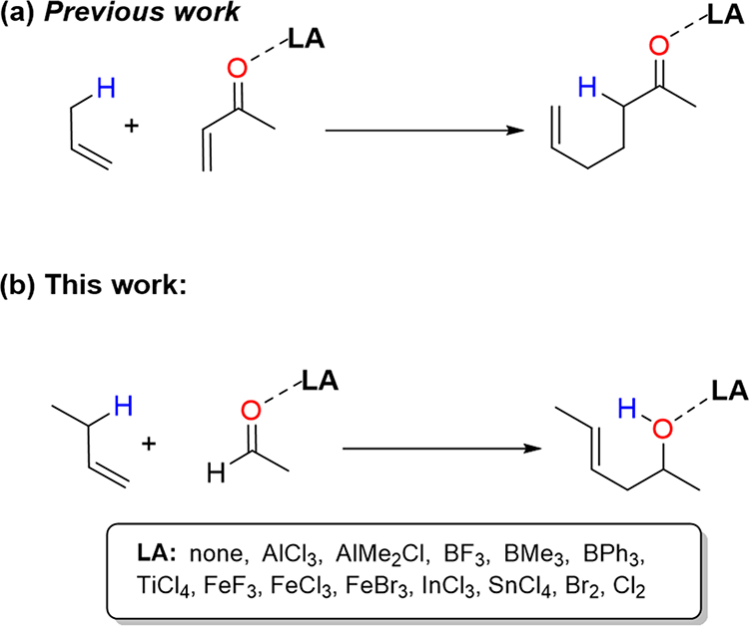
(a) Previous Work on Lewis Acid-Catalyzed Ene Reactions and (b) Carbonyl-Ene Reactions Considered in This Work

Typically, this remarkable acceleration is rationalized in terms of the stabilization of the LUMO of the enophile upon binding to the Lewis acid, which leads to a more favorable HOMO(ene)–LUMO(enophile) interaction.^10^ However, we recently found that this so-called LUMO-lowering concept in catalysis is rather incomplete for related Lewis acid-catalyzed Diels–Alder cycloaddition reactions.^11^ Instead, a remarkable decrease in the Pauli repulsion between the key occupied π-molecular orbitals and not an enhancement of orbital interactions constitutes the actual physical mechanism behind the acceleration induced by Lewis acids. This so-called *Pauli repulsion lowering concept*(12) seems general as it also operates in related Diels–Alder cycloaddition reactions where the catalyst establishes noncovalent interactions (hydrogen,^13^ halogen,^14^ or chalcogen bonds^15^) with the dienophile and even in slightly related dihalogen-catalyzed Michael-addition reactions^16^ and iminium-ion-catalyzed cycloadditions.^17^ The reduction in Pauli repulsion also plays a significant role in controlling the reactivity of uncatalyzed azadiene inverse-electron-demand Diels–Alder cycloadditions^18^ and even transition-metal-catalyzed reactions.^19^ This mechanism is also behind the acceleration observed in Lewis acid-catalyzed ene reactions involving α,β-unsaturated carbonyl compounds as enophiles (i.e., a parent ene reaction where the enophile is an alkene attached to an electron-withdrawing carbonyl group) as recently reported by Hamlin and co-workers (Scheme 2a).^20^ Despite this, it is unclear whether this Pauli repulsion lowering mechanism is also behind the acceleration induced by Lewis acids in carbonyl-ene reactions, where the carbonyl group is directly involved in the transformation, and therefore, the influence of the Lewis acid should be much more pronounced. This prompted us to carry out a detailed computational exploration of the factors governing the catalysis in these fundamental reactions. To this end, the intermolecular reaction involving 1-butene and acetaldehyde promoted by a wide variety of Lewis acids has been selected (Scheme 2b) and analyzed by applying the combination of the activation strain model (ASM)^21^ of reactivity with the energy decomposition analysis (EDA)^22^ method. In addition, issues such as the impact of aromaticity of the corresponding transition states (TSs) and the synchronicity on this particular process shall be also explored in detail.

## Results and Discussion

**Figure 1:**
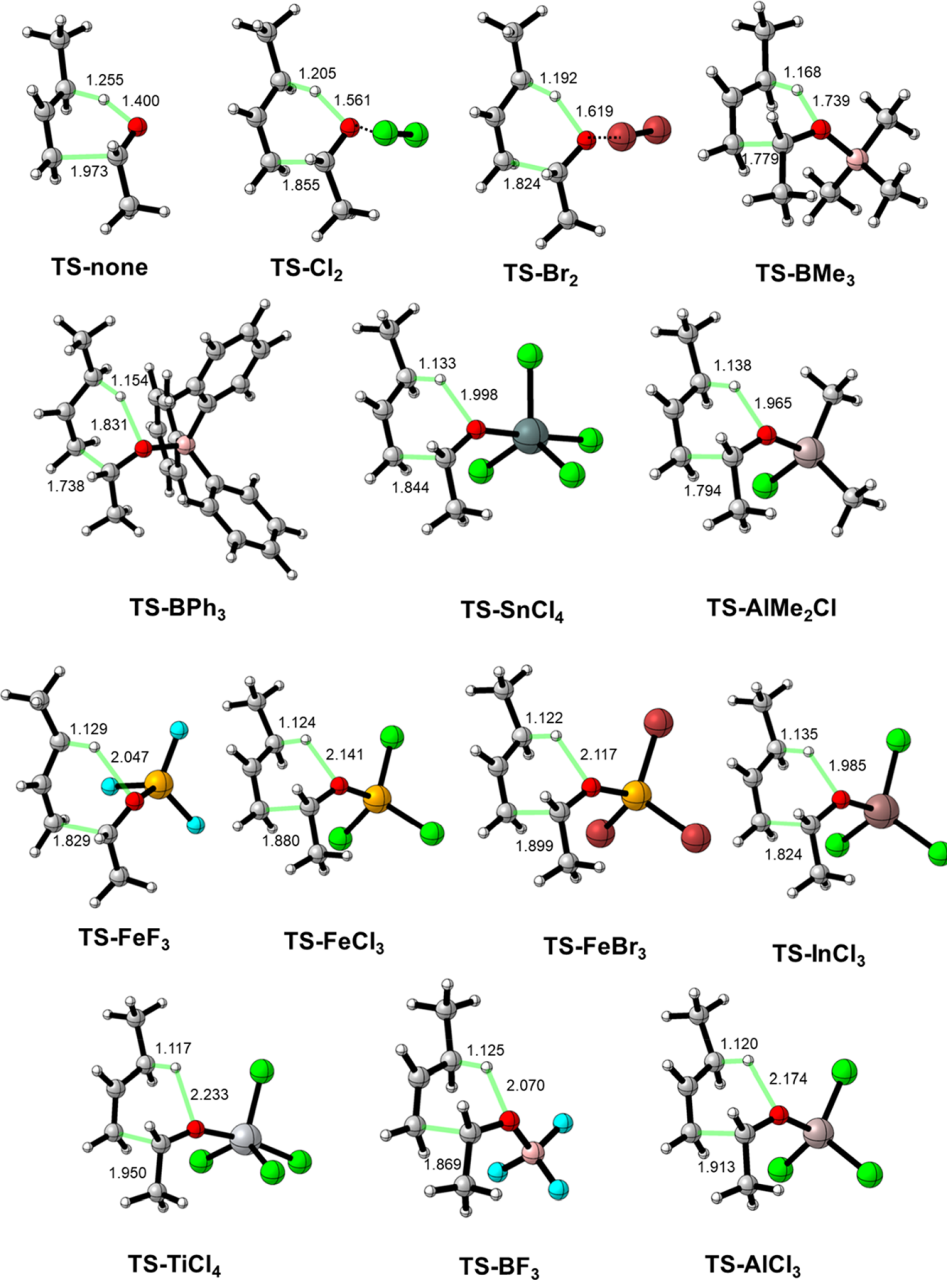
Optimized TSs involved in the considered carbonyl-ene reactions. Bond distances are given in angstroms. All data have been computed at the PCM(dichloromethane)-ωB97xD/def2-SVP level.

We first focused on the parent uncatalyzed reaction involving 1-butene and acetaldehyde and the corresponding Lewis acid-catalyzed processes. Our calculations (PCM(dichloromethane)-ωB97xD/def2-TZVP//PCM(dichloromethane)-ωB97xD/def2-SVP level) indicate that in all cases, the reaction proceeds concertedly via an asynchronous, six-membered TS, which leads to the exergonic formation of the respective (*E*)-hex-4-en-2-ol (see Figure 1). Despite this, note that under different conditions (for instance, 2 equiv of Lewis acid reagent or change in the Lewis acid), a stepwise mechanism may operate.^7,8^ As expected, the barrier height computed for the uncatalyzed reaction is relatively high (Δ*G*^≠^ = 44.1 kcal/mol), which agrees with previous calculations^23,24^ and the available experimental data (see above) for related ene reactions. At variance, the analogous LA-catalyzed reactions exhibit lower activation barriers that systematically decrease with the relative Lewis acidity of the catalyst. For instance, whereas weak Lewis acids, such as Cl_2_ or Br_2_, bonded to the carbonyl group through weak halogen bonds, lead only to a slight reduction in the barrier (Δ*G*^≠^ = 43.8 and 41.7 kcal/mol, respectively), strong LAs such as BF_3_ or AlCl_3_ lead to markedly lower barriers (Δ*G*^≠^ = 22.4 and 19.3 kcal/mol, respectively, see Table 1). Therefore, a remarkable reduction of the barrier of up to ca. 25 kcal/mol can be achieved in the LA-mediated carbonyl-ene reactions, which is fully consistent with the temperatures used experimentally (see above). Our calculations confirm that the effect of the Lewis acid is much more significant than when placed remotely (Scheme 2a), where a much lower reduction of the barrier (up to 12 kcal/mol for AlCl_3_) was found.^20^ Similar values and reactivity trends were found when considering the formation of the (*Z*)-hex-4-en-2-ol isomer (see Table S1 in the Supporting Information).

**Table 1:** Computed Activation and Reaction Energies (in kcal/mol), Synchronicity (*S*_y_), and NICS(3,+1) Values in the Corresponding TSs (in ppm) of the Considered Carbonyl-Ene Reactions

catalyst	Δ*G*^≠^^a^	Δ*E*^≠^^a^	Δ*G*_R_^b^	Δ*E*_R_^b^	*S*_y_	NICS(3,+1)
none	44.1	31.3	–2.6	–14.7	0.87	–19.9
Cl_2_	43.8	28.7	–1.9	–16.1	0.72	–16.9
Br_2_	41.7	26.8	–2.9	–16.6	0.68	–15.9
BMe_3_	34.8	19.8	–4.1	–18.5	0.62	–13.5
BPh_3_	28.0	14.1	–6.0	–19.6	0.58	–11.2
SnCl_4_	27.4	13.8	–3.4	–17.8	0.61	–9.9
AlMe_2_Cl	25.8	11.6	–6.1	–19.8	0.59	–10.3
FeF_3_	26.4	11.6	–3.2	–18.3	0.60	–9.6
FeCl_3_	24.0	9.5	–3.8	–18.4	0.58	–8.2
FeBr_3_	21.8	7.6	–5.7	–20.0	0.58	–8.0
InCl_3_	24.7	10.4	–6.7	–20.2	0.61	–10.5
TiCl_4_	24.0	10.2	–3.8	–17.9	0.55	–6.5
BF_3_	22.4	9.2	–5.9	–18.8	0.58	–8.8
AlCl_3_	19.3	6.1	–5.7	–19.0	0.56	–7.8

a Activation barriers computed as Δ*E*^≠^ = E(TS) – *E*(1-butene) – *E*(aldehyde).

b Reaction energies computed as Δ*E*_R_ = *E*((*E*)-hex-4-en-2-ol) – *E*(1-butene) – *E*(aldehyde). All data have been computed at the PCM(dichloromethane)-ωB97xD/def2-TZVPP//PCM(dichloromethane)-ωB97xD/def2-SVP level.

**Figure 2:**
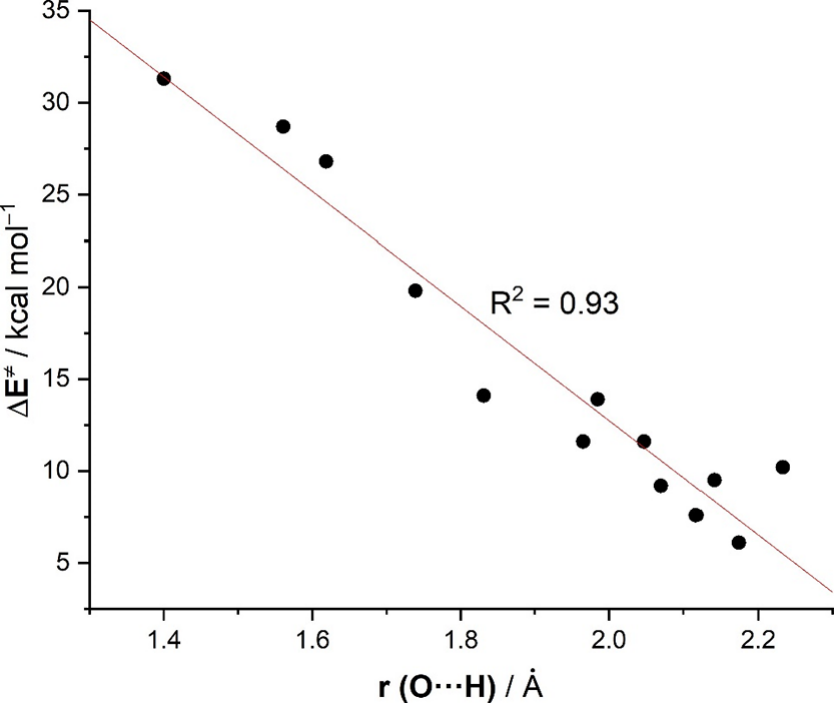
Correlation between the computed activation barriers (Δ*E*^≠^) and O···H bond-forming distances in the TSs associated with the considered carbonyl-ene reactions.

Closer inspection of the optimized geometries of the TSs (Figure 1) indicates that although the formation of the new C–C and O–H bonds is accompanied by the C–H bond rupture, the LA-catalyzed reactions are much more asynchronous than the parent uncatalyzed reaction. This is confirmed by the calculation of the synchronicity (*S*_y_) of the process (see Computational Details section), which shows that whereas the uncatalyzed reaction presents a high *S*_y_ value of 0.87 (close to the value for a synchronous process, *S*_y_ = 1), the LA-mediated reactions exhibit much lower values (*S*_y_ ranging from 0.72 to 0.55), which, for those cases involving strong Lewis acids (such as AlCl_3_), are actually in the limit for a concerted process. This asynchronicity is manifested in both the C···C bond-forming distance, which becomes shorter in the catalyzed reactions and particularly, in the O···H bond-forming distance, which becomes much longer. Interestingly, there appears to be a correlation between the computed barriers and these geometrical parameters in the sense that the transformation is kinetically easier (i.e., proceeds with a lower barrier) when the O···H bond-forming distance is longer. Indeed, when plotting both parameters, a very good linear relationship was found (correlation coefficient of 0.93, Figure 2), which suggests that these carbonyl-ene reactions follow the Hammond–Leffer postulate.^25^

**Figure 3:**
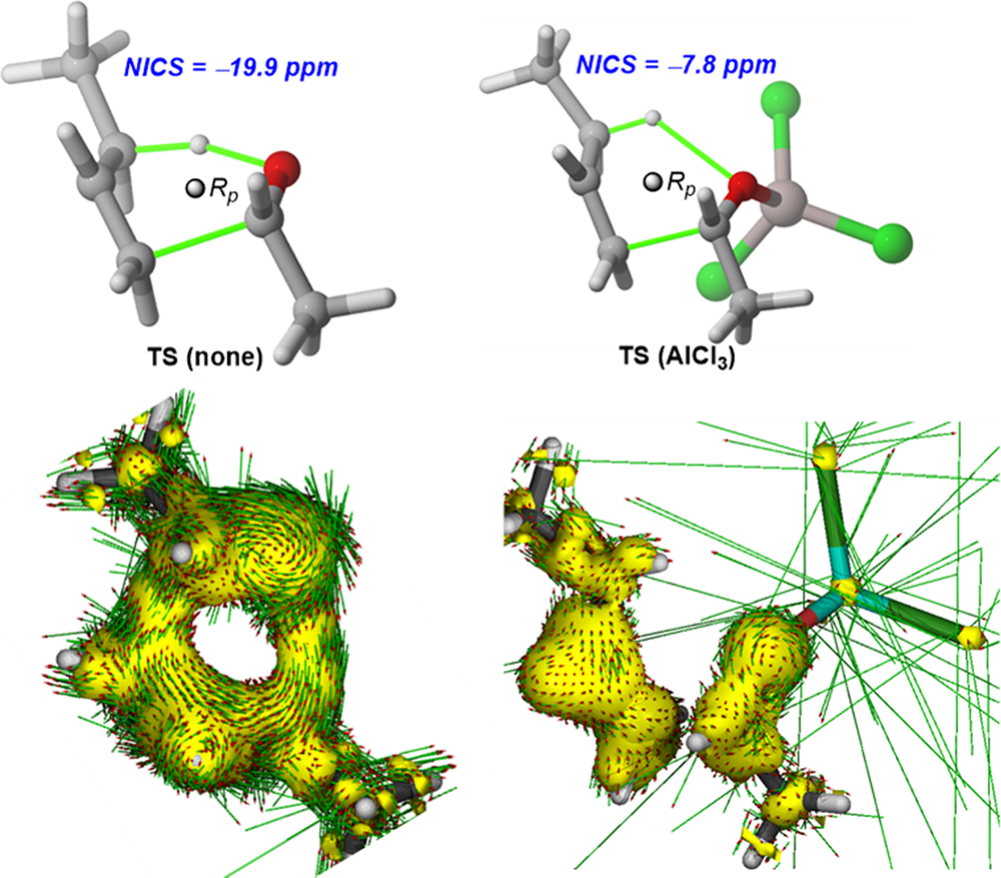
NICS values and AICD plots for the TSs associated with the uncatalyzed carbonyl-ene reaction (left) and the analogous reaction catalyzed by AlCl_3_ (right).

Similar to other pericyclic reactions,^26^ including the parent ene reaction involving an alkene as an enophile,^24^ the transition structure for the uncatalyzed carbonyl-ene reaction can be considered as aromatic according to the negative nuclear independent chemical shift (NICS)^27^ value computed at the (3,+1) ring critical point (RCP)^28^ of the six-membered cycle (NICS(3,+1) = −19.9 ppm, see Table 1). This highly negative NICS value results from the involvement of the six [σ^2^ + π^2^ + π^2^] electrons in the concerted process, which approximately lie in the molecular plane leading to a remarkable diamagnetic shielding at the RCP. Indeed, the delocalization of these six electrons becomes evident when applying the anisotropy of the induced current density (AICD)^29^ method, which clearly shows a diatropic (i.e., clockwise vectors) current within the six-membered ring (Figure 3, left). Moreover, the in-plane aromatic^30^ nature of this TS is further supported by the variation of the NICS values along the *z*-axis perpendicular to the molecular plane, which shows the usual bell-shaped plot having a maximum NICS value at *z* = 0 Å (i.e., at the RCP, see Figure S1 in the Supporting Information).

Data in Table 1 also indicate that the analogous Lewis acid-catalyzed reactions also feature in-plane aromatic TSs. However, the computed NICS(3,+1) values are systematically lower (NICS ranging from −16.9 to −6.5 ppm) than that computed for the parent uncatalyzed reaction. Once again an evident relationship between the relative Lewis acidity of the catalyst and the computed aromaticity was found. This is not surprising because, as commented above, Lewis acids lead to more asynchronous TSs, which in turn results in a less efficient delocalization of the six electrons involved in the transformation and consequently, in a lower diamagnetic shielding at the corresponding RCP. This can be also confirmed by the visualization of the corresponding ring current by the AICD method, which clearly shows, for the reaction mediated by the strong Lewis acid AlCl_3_, an interrupted ring current that severely hampers the electronic delocalization in the transformation (Figure 3, right), which is reflected in the computed rather low NICS value.

**Figure 4:**
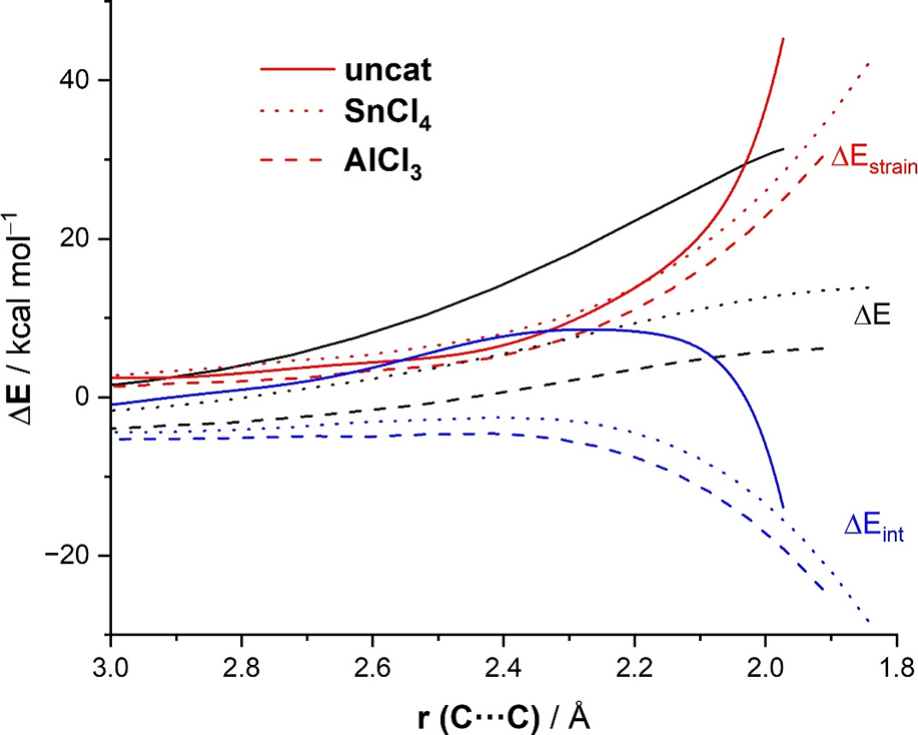
ASDs of the carbonyl-ene reactions between 1-butene and acetaldehyde (uncatalyzed, solid lines) and analogous catalyzed reactions by SnCl_4_ (dotted lines) and AlCl_3_ (dashed lines) projected onto the C···C bond-forming distance. All data have been computed at the PCM(dichloromethane)-ωB97xD/def2-TZVPP//PCM(dichloromethane)-ωB97xD/def2-SVP level.

Despite the clear impact of the Lewis acid on the (in-plane) aromaticity of the TSs, there is no straightforward, causal physical relationship between the above computed magnetic indicators and the energetics of the transformation. Indeed, one should expect that an increase in the aromaticity should result in a gain in stability and, therefore, in a lower barrier, which is not the case. A similar finding was observed in double-group transfer reactions, related pericyclic processes, which typically involve the concerted migration of two atoms or groups from one compound to another.^30d^ For this reason, we then applied the activation strain model (ASM)^21^ of reactivity to understand, in a quantitative manner, the factors leading to the acceleration induced by the Lewis acids in these carbonyl-ene reactions. To this end, we focused on the parent uncatalyzed reaction and the analogous processes mediated by the strong Lewis acid AlCl_3_ and a weaker acid, SnCl_4_ (Δ*E*^≠^ decreases in the order: 31.3 kcal/mol, **none** >13.8 kcal/mol, **SnCl_4_** >6.1 kcal/mol, **AlCl**_3_). The corresponding activation strain diagrams (ASDs) for these reactions, computed from the initial stages of the transformation up to the respective TSs and projected onto the C···C bond-forming distances,^31^ are shown in Figure 4. Data in Figure 4 indicate that the catalyzed reactions benefit from a less destabilizing strain energy (measured by the Δ*E*_strain_ term), in particular, at the TS region. The trend in Δ*E*_strain_ (**none** > **SnCl_4_** > **AlCl**_**3**_) can be directly ascribed to the extent of the asynchronicity (*S*_y_ decreases in the order: 0.87, **none** >0.61, **SnCl_4_** >0.56, **AlCl**_3_), since a higher asynchronicity implies that the energy penalty required by the reactants (mainly the alkene in this particular transformation) to reach the TS geometry is lower. Despite this, the main factor behind the remarkable reduction in the barrier height is a significant enhancement in the interaction energy between the increasingly deformed reactants. As depicted in Figure 4, the catalyzed reactions exhibit a much stronger Δ*E*_int_ along the entire reaction coordinate, and once again, the enhancement in Δ*E*_int_ is directly related to the relative Lewis acidity of the catalyst. Therefore, our ASM analysis indicates that the catalysis in these carbonyl-ene reactions originates mainly from an enhancement of the interaction between the deformed reactants and, also, from a less destabilizing geometrical distortion as a consequence of a greater asynchronicity (albeit to a lesser extent).

**Figure 5:**
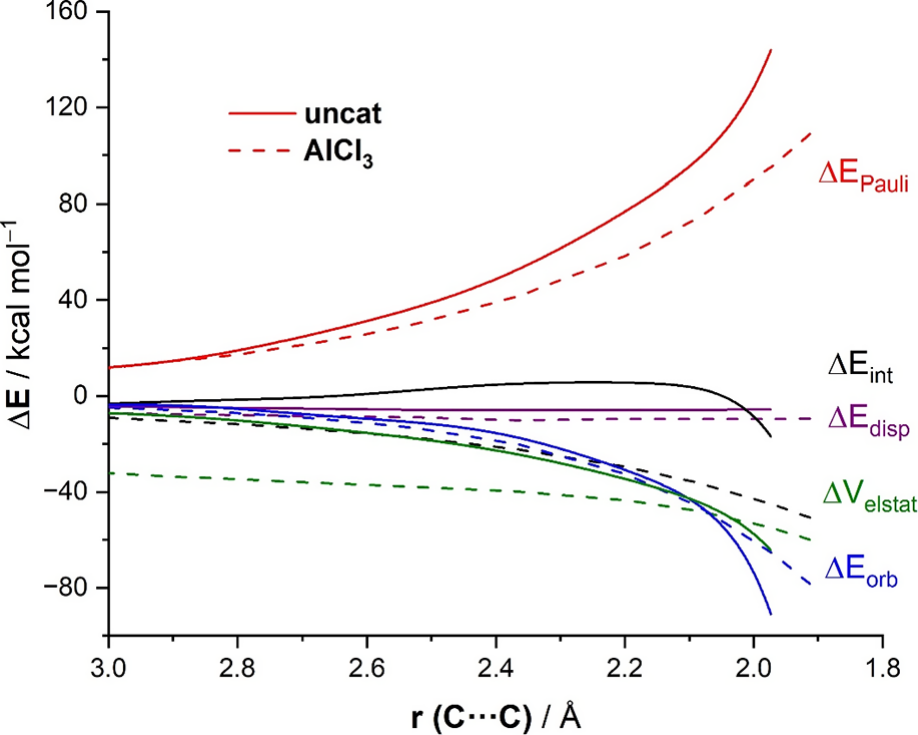
Energy decomposition analyses of the carbonyl-ene reactions between 1-butene and acetaldehyde (uncatalyzed, solid lines) and the analogous catalyzed reaction by AlCl_3_ (dotted lines) projected onto the C···C bond-forming distance. All data have been computed at the ZORA-ωB97xD/TZ2P//PCM(dichloromethane)-ωB97xD/def2-SVP level.

The factors behind the stronger interaction between the deformed reactants computed for the Lewis acid-mediated reactions discussed above can be analyzed using the energy decomposition analysis (EDA).^22^ The change in the EDA terms along the reaction coordinate for the extreme situations represented by the uncatalyzed and AlCl_3_-catalyzed carbonyl-ene reactions is graphically shown in Figure 5. At the TS region, the attractive electrostatic, Δ*V*_elstat_, and orbital, Δ*E*_orb_, interactions are similar or even slightly more stabilizing for the uncatalyzed reaction, which suggests that the stronger interaction computed for the AlCl_3_-catalyzed reaction does not derive from either the electrostatic attractions or the orbital interactions. At variance, data in Figure 5 indicate that the catalyzed reaction clearly exhibits a much less destabilizing Pauli repulsion practically along the entire transformation. For instance, the difference in this crucial term is ΔΔ*E*_Pauli_ = 38.0 kcal/mol (favoring the catalyzed reaction), when computed at the same consistent C···C bond-forming distance of 2.0 Å.^32^ This suggests that this significant reduction in the Pauli repulsion compensates the main attractive terms (Δ*V*_elstat_ and Δ*E*_orb_) and becomes the exclusive factor behind the enhanced interaction computed for the catalyzed reaction. Therefore, our calculations confirm that similar to other catalyzed pericyclic reactions,^11−15^ the origin of the acceleration induced by Lewis acids in these carbonyl-ene reactions, where the carbonyl group is directly involved in the transformation, can be also rationalized in terms of the *Pauli repulsion lowering concept*.^12^

**Figure 6:**
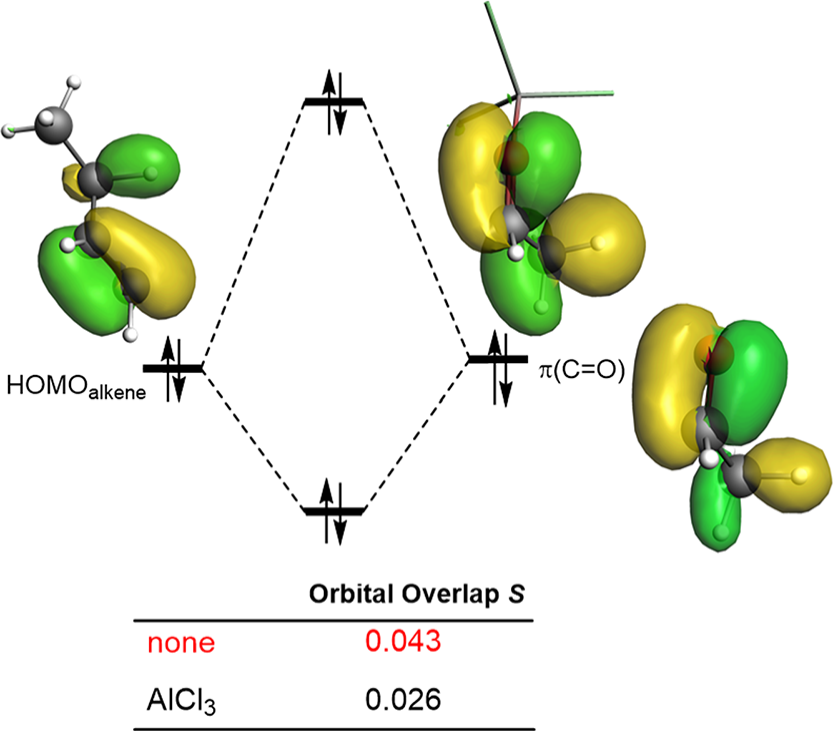
Schematic orbital interaction diagram of the main occupied orbital overlap involved in the carbonyl-ene reactions. All data have been computed at the ZORA-ωB97xD/TZ2P//PCM(dichloromethane)-ωB97xD/def2-SVP level.

We can further investigate the reasons leading to the reduction in the crucial Δ*E*_Pauli_ term for the Lewis acid-catalyzed Alder-ene reactions by performing a Kohn–Sham molecular orbital analysis. It is found that the four-electron interaction between the doubly occupied HOMO of the alkene (mainly centered in the reactive C=C and C–H bonds) and the filled π–(C=O) molecular orbital of the enophile (Figure 6) constitutes the main contributor to the Pauli repulsion. At a C···C bond-forming distance of 2.0 Å,^32^ the corresponding HOMO(alkene)-π(C=O) orbital overlap (*S*) is much larger, and therefore, much more destabilizing for the uncatalyzed reaction (*S* = 0.043 vs *S* = 0.026). This reduction in the overlap for the catalyzed reaction results from the polarization, induced by the Lewis acid, of the occupied π–(C=O) molecular orbital of the enophile. This translates into a noticeable depopulation in the reactive carbon atom of the carbonyl in the acetaldehyde-AlCl_3_ complex as compared to the parent CH_3_CHO as viewed from the increase of the positive charge at this carbon atom (computed NBO-charge of +0.46 in CH_3_CHO vs +0.59 in CH_3_CHO–AlCl_3_). Not surprisingly, the extent of this polarization of the key π(C=O) orbital directly depends on the relative Lewis acidity of the catalyst. For instance, the corresponding charge at the reactive carbon atom in the weak Lewis acid complex CH_3_CHO–Cl_2_ (+0.48) resembles that of the parent acetaldehyde, whereas the stronger CH_3_CHO–SnCl_4_ complex exhibits a more positive value (+0.54), which of course is lower than that computed for their AlCl_3_-counterpart. Although the decrease in the charges is small compared to the significant changes in the Pauli repulsion, these small changes in charges translated into a dramatic lowering of Pauli repulsion. Therefore, it can be concluded that the polarization of the key π(C=O) orbital of the carbonyl group induced by the Lewis acid, which is reflected in a clear reduction of Δ*E*_Pauli_, is the ultimate factor controlling the catalysis in these carbonyl-ene reactions.

**Figure 7:**
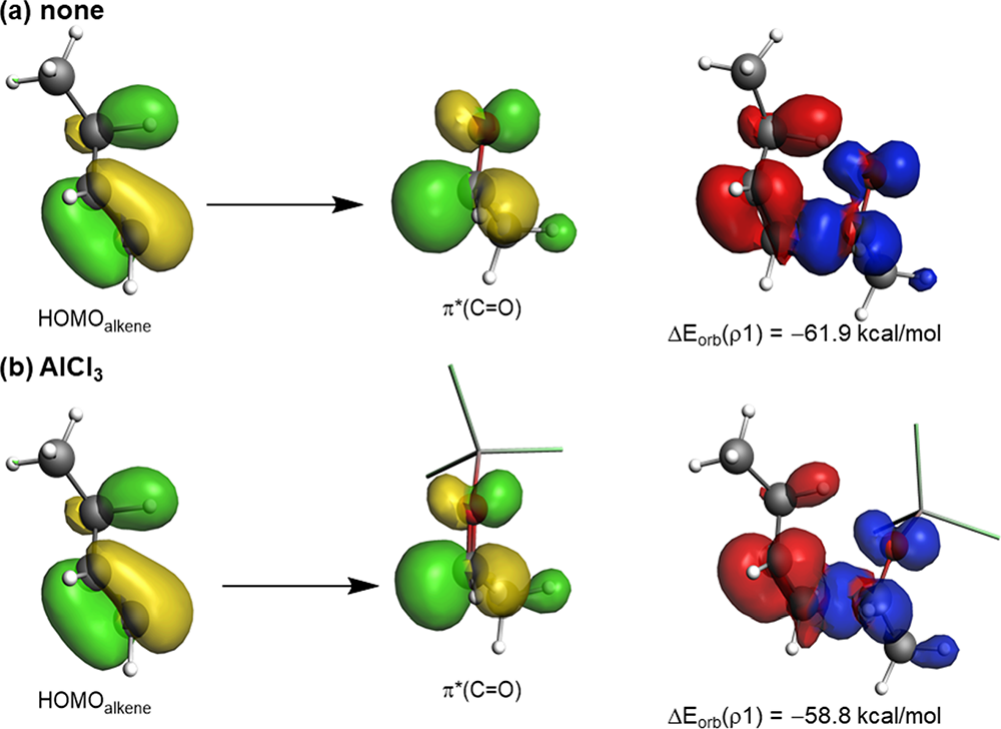
Plot of the deformation densities Δρ and associated molecular orbitals together with the corresponding stabilization energies Δ*E*(ρ) involved in the parent uncatalyzed carbonyl-ene reaction (a) and its AlCl_3_-catalyzed counterpart (b). Charge flows in the red → blue direction. All data have been computed at the ZORA-ωB97xD/TZ2P//PCM(dichloromethane)-ωB97xD/def2-SVP level.

Finally, we were curious to understand why the catalyzed reactions exhibit weaker orbital interactions although the corresponding enophile features a more stabilized π*(C=O) molecular orbital (for instance, 0.05 eV vs −0.73 eV, for CH_3_CHO and CH_3_CHO–AlCl_3_, respectively). According to the frontier molecular orbital (FMO) theory, this would result in a more favorable HOMO(alkene)−π*–(C=O) interaction and therefore, in stronger orbital interactions. However, our calculations indicate that the trend in the Δ*E*_orb_ term is the opposite, i.e., slightly stronger for the uncatalyzed reaction (see above). One plausible explanation would be once again related to the marked asynchronicity of the catalyzed reactions in the sense that the longer H···O bond-forming distance should result in a less favorable HOMO−π* overlap. Indeed, our calculations confirm this hypothesis as viewed from the computed smaller overlap for the AlCl_3_-mediated reaction (*S* = 0.21) as compared to the analogous uncatalyzed process (*S* = 0.24). Moreover, we also applied the natural orbital for chemical valance (NOCV)^33^ extension of the EDA method, which allows not only the visualization but also the quantification of the main orbital interactions involved in the total Δ*E*_orb_ term. As expected, the HOMO(alkene) → π*–(C=O) interaction (denoted as ρ1) method constitutes the main orbital interaction between the reactants, contributing ca. 90% to the total Δ*E*_orb_ term (see Figure 7). Interestingly, and although the π*(C=O) molecular orbital becomes stabilized in the catalyzed system, the strength of this interaction is clearly higher (i.e., stronger) in the uncatalyzed reaction than in the AlCl_3_-catalyzed reaction (see stabilizing energies at a C···C bond-forming distance of 2.0 Å in Figure 7), which, in addition to the computed larger overlap, derives in the stronger orbital interactions Δ*E*_orb_ computed for the uncatalyzed reaction. This result confirms that one should be cautious when using arguments solely derived from the energy of the FMO of the reactants, particularly in catalyzed pericyclic reactions.

## Conclusions

From the above computational results, it is found that the binding of a Lewis acid to the carbonyl group directly involved in the considered carbonyl-ene reactions results in a remarkable reduction of the activation barrier (up to 25 kcal/mol) in comparison to the parent uncatalyzed reaction. Although the process occurs concertedly, via a six-membered, in-plane aromatic TS, the catalyzed reactions are much more asynchronous, being the extent of the asynchronicity directly related to the relative Lewis acidity of the catalyst. Interestingly, there exists a clear correlation between the barrier heights and the synchronicity in the sense that more asynchronous processes (i.e., mediated by stronger Lewis acids) are associated with lower barriers. In addition, the TSs for the catalyzed carbonyl-ene reactions are less aromatic than their analogous uncatalyzed saddle points. Moreover, their relative aromaticity strength also correlates with the asynchronicity of the process. Although the key π*(C=O) molecular orbital of the enophile becomes stabilized in the enophile-LA complexes, the observed acceleration in the catalyzed processes does not result from a more favorable HOMO(alkene)−π*(C=O) interaction, as traditionally viewed. Instead, our ASM-EDA calculations indicate that the catalysis finds its origin in a lower strain energy (as a consequence of the induced asynchronicity) and, more importantly, in a stronger interaction between the deformed reactants, which mainly results from a remarkable decrease of the destabilizing Pauli repulsion between the key π-molecular orbitals of the reagents. This confirms the general applicability of the *Pauli repulsion lowering concept* in catalysis.

## Experimental Section

### Computational Details

Geometry optimizations were performed without symmetry constraints using the Gaussian-16 (RevB.01)^34^ suite of programs at the dispersion-corrected ωB97xD^35^/def2-SVP^36^ level. Solvent effects (solvent = dichloromethane) were considered during the geometry optimizations by means of the polarization continuum model (PCM) method.^37^ This level is denoted as the PCM(dichloromethane)-ωB97xD/def2-SVP level. Reactants and adducts were characterized by frequency calculations and have positive definite Hessian matrices. TSs show only one negative eigenvalue in their diagonalized force constant matrices, and their associated eigenvectors were confirmed to correspond to the motion along the reaction coordinate under consideration using the intrinsic reaction coordinate (IRC) method.^38^ Natural bond order (NBO) calculations were performed with the NBO6.0 program^39^ at the PCM(dichloromethane)-ωB97xD/def2-SVP level. Additional single-point energy refinements were carried out at same DFT level using the much larger triple-ζ def2-TZVPP basis set.^36^ This level is denoted as the PCM(dichloromethane)-ωB97xD/def2-TZVPP//PCM(dichloromethane)-ωB97xD/def2-SVP level.

The aromaticity of the transition structures was assessed by the computation of the NICS^27^ values using the gauge invariant atomic orbital (GIAO) method^40^ at the B3LYP^41^/def2-SVP//PCM(dichloromethane)-ωB97xD/def2-SVP level. Ring currents were computed using the AICD method.^29^

The synchronicity^42,43^ of the reactions was quantified following a previously described approach.^44,45^ For a concerted reaction, synchronicity is defined as^46^
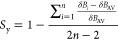
where *n* is the number of bonds involved in the reaction (in this case, *n* = 6) and δ*B_i_* measures the relative variation of a given bond index *B_i_* at the TS, according to the following formula:
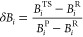
where the superscripts R and P refer to the reactants and the product, respectively. The average value of δ*B_i_*, denoted as δ*B*_AV_ is therefore
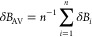
The Wiberg bond indices *B_i_* were computed using the NBO method.^39^

#### ASM of Reactivity and EDA

Within the ASM method,^21^ the potential energy surface Δ*E*(ζ) is decomposed along the reaction coordinate, ζ, into two contributions, the strain Δ*E*_strain_(ζ) associated with the deformation (or distortion) required by the individual reactants during the process and the interaction Δ*E*_int_(ζ) between these increasingly deformed reactants:
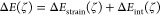


The EDA method^22^ can be used to further decompose the interaction energy into the following chemically meaningful terms:
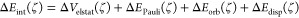


The term Δ*V*_elstat_ corresponds to the classical electrostatic interaction between the unperturbed charge distributions of the deformed reactants and is usually attractive. The Pauli repulsion Δ*E*_Pauli_ comprises the destabilizing interactions between occupied orbitals and is responsible for any steric repulsion. The orbital interaction Δ*E*_orb_ accounts for bond pair formation, charge transfer (interactions between occupied orbitals on one moiety with unoccupied orbitals on the other, including HOMO–LUMO interactions), and polarization (empty-occupied orbital mixing on one fragment due to the presence of another fragment). Finally, the Δ*E*_disp_ takes into account those interactions coming from dispersion forces. Moreover, the NOCV (natural orbital for chemical valence)^33^ extension of the EDA method has been also used to further partition the Δ*E*_orb_ term. The EDA-NOCV approach provides pairwise energy contributions for each pair of interacting orbitals to the total bond energy. The ASM-EDA(NOCV) approach has proven to provide detailed quantitative insight into the ultimate factors controlling fundamental processes in organic, main group, and organometallic chemistry.^47^

The program package ADF^48^ was used for EDA calculations using the optimized PCM(dichloromethane)-ωB97xD/def2-SVP geometries at the same DFT level in conjunction with a triple-ζ-quality basis set using uncontracted Slater-type orbitals (STOs) augmented by two sets of polarization functions with a frozen-core approximation for the core electrons.^49^ Auxiliary sets of s, p, d, f, and g STOs were used to fit the molecular densities and to represent the Coulomb and exchange potentials accurately in each SCF cycle.^50^ Scalar relativistic effects were incorporated by applying the zeroth-order regular approximation (ZORA).^51^ This level of theory is denoted as ZORA-ωB97xD/TZ2P//PCM(dichloromethane)-ωB97xD/def2-SVP.

## Data Availability

The data underlying this study are available in the published article and its Supporting Information.

## Supporting Information Available

Evolution of the NICS values (Figure S1), activation and reaction energies, synchronicity, and NICS(3,+1) values (Table S1), and Cartesian coordinates (in Å) and total energies of all the stationary points discussed in the text (PDF)
