# Supplementary material for: Lewis Acid-Catalyzed Carbonyl-Ene Reaction: Interplay between Aromaticity, Synchronicity, and Pauli Repulsion
Source: J Org Chem. 2023 Jul 24;88(15):11102–10. doi: 10.1021/acs.joc.3c01059 (PMC10407925; doi:10.1021/acs.joc.3c01059)
Supplement: Supplementary file 1 — jo3c01059_si_001.pdf [file jo3c01059_si_001.pdf]

Lewis-acid Catalyzed Carbonyl-ene Reaction: Interplay between Aromaticity, Synchronicity and Pauli Repulsion

Humberto A. Rodríguez,[≠,‡] Daniel A. Cruz,[≠] Juan I. Padrón,[‡] and Israel Fernández[≠,*]

[‡]Instituto de Productos Naturales y Agrobiología, Consejo Superior de Investigaciones Científicas (IPNA-CSIC), Avda. Astrofísico Francisco Sánchez 3, 38206 La Laguna, Tenerife, Islas Canarias, Spain

[≠]Departamento de Química Orgánica I and Centro de Innovación en Química Avanzada (ORFEO-CINQA), Facultad de Ciencias Químicas, Universidad Complutense de Madrid, 28040-Madrid, Spain.

israel@quim.ucm.es

Contents:

1. Table S1 and Figure S1……………………………………………………S2
2. Cartesian coordiantes…………………………………………………….S3
3. Synchronicity values……………………………………………………..S35

Table S1. Activation and reaction energies (in kcal/mo1), synchronicity (S_y) and NICS(3,+1) values in the corresponding transition states (in ppm) of the considered carbonyl-ene reactions leading to (*Z*)-hex-4-en-2-ol.

Catalyst	$\Delta G^{\neq a}$	$\Delta E^{\neq a}$	$\Delta G_R{}^b$	$\Delta E_R{}^b$	S_y (E)	NICS(3,+1)
none	44.7	32.1	-1.4	-13.8	0.88	-20.3
Cl_2	44.1	29.2	-1.4	-15.0	0.78	-19.0
Br_2	42.3	27.6	-1.7	-15.4	0.74	-18.1
BMe_3	37.9	22.5	-3.4	-17.2	0.66	-14.6
BPh_3	30.9	16.9	-6.9	-20.2	0.62	-11.3
$SnCl_4$	28.9	14.2	-4.3	-17.9	0.60	-9.4
$AlMe_2Cl$	28.1	13.1	-5.0	-19.2	0.53	-12.4
FeF_3	27.4	13.3	-3.6	-17.6	0.59	-11.4
$FeCl_3$	24.4	9.6	-3.4	-17.4	0.56	-7.7
$FeBr_3$	22.7	7.5	-5.3	-19.6	0.55	-7.0
$InCl_3$	25.6	10.7	-5.6	-20.0	0.62	-10.7
$TiCl_4$	24.2	10.1	-4.9	-18.6	0.52	-5.4
BF_3	23.5	9.6	-6.4	-18.9	0.58	-8.6
$AlCl_3$	20.1	6.0	-5.1	-18.8	0.54	-7.0

[a] Activation barriers computed as $\Delta E^{\neq}$ = E(**TS**) – E(1-butene) – E(aldehyde). [b] Reaction energies computed as ΔE_R = E((*Z*)-hex-4-en-2-ol) – E(1-butene) – E(aldehyde). All data have been computed at the PCM(dichloromethane)-ωB97xD/def2-TZVPP//PCM(dichloromethane)-ωB97xD/def2-SVP level.

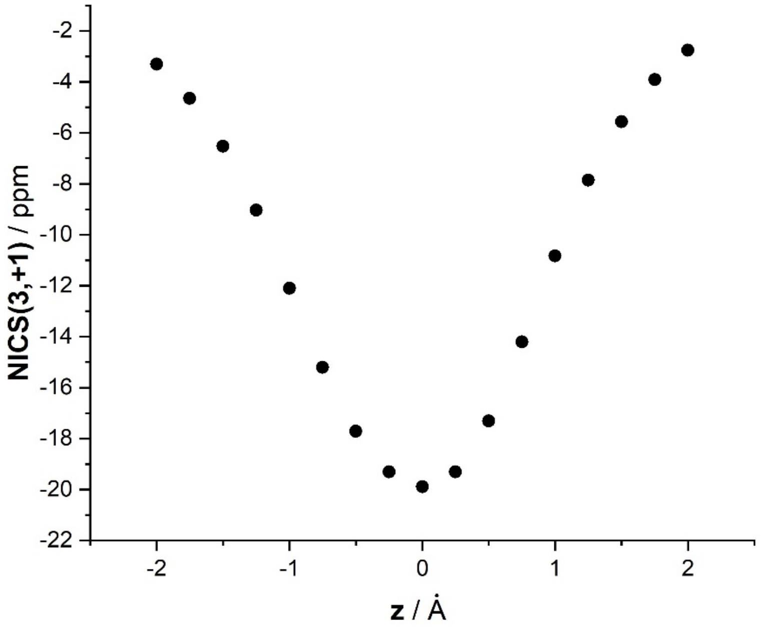

Figure S1. Evolution of the NICS values along the z axis perpendicular to the molecular plane of the parent uncatalyzed transition state **TS-none**. Data computed at the B3LYP/def2-SVP//PCM(dichloromethane)-ωB97xD/def2-SVP level.

Cartesian coordinates (in Å) and total energies (in a.u., noncorrected ZVPE included) of all the stationary points discussed in the text. All calculations have been performed at the PCM(DCM)-ωb97xd/def2- SVP level. ***SP*** denotes values computed at the PCM(DCM)-ωb97xd/def2-TZVP level//PCM(DCM)-ωb97xd/def2-SVP level level.

But-1-ene
E = -157.121255
G = -157.148846
N_{imag} = 0
SP = -157.230127

C	-1.849582000	0.015747000	-0.276402000
C	-0.716474000	-0.292370000	0.337862000
C	0.538083000	0.524344000	0.302396000
H	-1.945527000	0.924634000	-0.860255000
H	-2.719559000	-0.625626000	-0.215848000
H	0.364535000	1.439650000	-0.267069000
H	0.792238000	0.828239000	1.322013000
H	-0.661801000	-1.215611000	0.910127000
C	1.715552000	-0.248985000	-0.290939000
H	1.900727000	-1.167910000	0.268060000
H	1.516263000	-0.523443000	-1.327642000
H	2.627647000	0.347651000	-0.266889000

Acetaldehyde
E = -153.793463
G = -153.818404
N_{imag} = 0
SP = -153.849202

C	-0.228368000	0.395526000	-0.000048000
H	-0.303825000	1.499276000	0.000068000
O	-1.229956000	-0.274448000	0.000004000
C	1.161399000	-0.149727000	-0.000032000
H	1.691818000	0.228383000	0.876604000
H	1.160560000	-1.236997000	-0.000977000
H	1.692904000	0.230128000	-0.875248000

TS (*E*)-hex-4-en-2-ol: without catalyst
E = -310.517082
G = -310.549208
N_{imag} = -1063.3822 cm^{-1}
SP = -311.029446

C	1.377291000	-0.317154000	-0.454259000
C	0.473940000	1.348733000	0.095747000
C	-0.827990000	1.020366000	-0.259255000
C	-1.525142000	-0.008865000	0.424555000
H	0.738455000	1.292134000	1.156537000
H	1.000691000	2.120436000	-0.471885000
H	-1.180004000	1.303207000	-1.260251000
H	-1.360575000	-0.025582000	1.512428000
H	1.463877000	-0.055362000	-1.530562000
H	-0.646214000	-0.841884000	0.095348000
O	0.645030000	-1.322576000	-0.152560000
C	2.686826000	-0.152356000	0.299891000
H	2.526727000	-0.299600000	1.377608000
H	3.385916000	-0.928773000	-0.049381000

H	3.150647000	0.828817000	0.126181000
C	-2.917124000	-0.424749000	-0.004666000
H	-3.154772000	-1.433959000	0.360676000
H	-3.682245000	0.260072000	0.392845000
H	-3.009546000	-0.434750000	-1.101145000

TS (*Z*)-hex-4-en-2-ol: without catalyst
***E* =** -310.516140
***G* =** -310.548564
N_{imag} = -1099.0847 cm^{-1}
***SP* =** -311.028158

C	1.337333000	0.004266000	-0.478287000
C	0.184274000	1.253204000	0.551901000
C	-0.937574000	1.205779000	-0.266396000
C	-1.700383000	0.019885000	-0.421705000
H	0.171119000	0.693677000	1.491267000
H	0.773940000	2.173557000	0.579853000
H	-1.021541000	1.958730000	-1.059011000
H	1.735661000	0.778063000	-1.167866000
H	-0.695360000	-0.535907000	-0.948956000
O	0.631117000	-0.930352000	-0.996394000
C	2.319504000	-0.385234000	0.613007000
H	1.834579000	-1.043823000	1.347782000
H	3.147114000	-0.944800000	0.148527000
H	2.743434000	0.490001000	1.124920000
C	-2.021033000	-0.896357000	0.748083000
H	-2.801803000	-0.461845000	1.392057000
H	-2.389306000	-1.865220000	0.382203000
H	-1.136638000	-1.097447000	1.367735000
H	-2.502853000	0.088576000	-1.166980000

(*E*)-hex-4-en-2-ol
***E* =** -310.583757
***G* =** -310.616927
N_{imag} = 0
***SP* =** -311.102821

C	1.475272000	0.076391000	0.358434000
C	0.451094000	-0.937499000	-0.175782000
C	-0.933071000	-0.640071000	0.322683000
C	-1.908504000	-0.095741000	-0.413699000
H	0.474824000	-0.907720000	-1.278087000
H	0.759674000	-1.948734000	0.136940000
H	-1.122129000	-0.839998000	1.386316000
H	-1.708787000	0.103407000	-1.475215000
H	1.477845000	0.003393000	1.466463000
H	0.181244000	1.488215000	0.110883000
O	1.129919000	1.386491000	-0.036078000
C	2.879592000	-0.202539000	-0.144469000
H	2.905016000	-0.166072000	-1.244644000
H	3.580994000	0.551687000	0.240769000
H	3.222486000	-1.194888000	0.182557000
C	-3.277408000	0.257942000	0.079914000
H	-3.487739000	1.327625000	-0.079716000
H	-4.051340000	-0.299867000	-0.471792000
H	-3.393291000	0.040117000	1.151659000

(*Z*)-hex-4-en-2-ol
***E* =** -310.582178
***G* =** -310.615004
N_{imag} = 0

***SP* =** -311.101248

C	1.333785000	0.106774000	-0.333813000
C	0.287386000	0.906333000	0.461096000
C	-0.982027000	1.111469000	-0.318491000
C	-2.078016000	0.340196000	-0.279817000
H	0.093633000	0.375971000	1.407166000
H	0.720833000	1.884745000	0.722090000
H	-0.982190000	1.954924000	-1.018894000
H	1.579831000	0.682464000	-1.250136000
H	-0.009442000	-1.026656000	-1.137968000
O	0.834008000	-1.165072000	-0.690066000
C	2.611006000	-0.108754000	0.456419000
H	2.395691000	-0.653451000	1.388768000
H	3.327592000	-0.700010000	-0.131405000
H	3.078692000	0.852629000	0.712631000
C	-2.307101000	-0.870488000	0.576633000
H	-3.070566000	-0.667411000	1.345607000
H	-2.690943000	-1.703618000	-0.032830000
H	-1.391104000	-1.214936000	1.073772000
H	-2.914293000	0.622747000	-0.930439000

Acetaldehyde-$AlCl_3$

***E* =** -1777.125422

***G* =** -1777.162791

N_{imag} = 0

***SP* =** -1777.188828

C	-2.045095000	0.022406000	-1.365963000
H	-2.482072000	0.060616000	-2.367000000
O	-0.816306000	0.019146000	-1.308447000
C	-2.958508000	-0.006093000	-0.220300000
H	-2.456225000	-0.126521000	0.734507000
H	-3.524262000	0.931099000	-0.237932000
H	-3.690888000	-0.799553000	-0.384153000
Al	0.472154000	-0.001817000	0.045999000
Cl	2.306019000	0.087781000	-1.009141000
Cl	0.052858000	1.727775000	1.208349000
Cl	0.145094000	-1.832795000	1.073836000

TS (*E*)-hex-4-en-2-ol: $AlCl_3$

***E* =** -1933.331956

***G* =** -1933.375958

N_{imag} = -300.8111 cm^{-1}

***SP* =** -1934.409191

C	-0.613565000	1.703102000	-0.905376000
C	-2.028200000	1.750385000	0.381529000
C	-2.836708000	0.759209000	-0.136622000
C	-2.605806000	-0.669270000	0.077074000
H	-1.434538000	1.515791000	1.271037000
H	-2.360818000	2.785146000	0.270493000
H	-3.600164000	1.044638000	-0.872569000
H	-2.178547000	-0.850436000	1.075432000
H	-1.177908000	1.960976000	-1.813458000
H	-1.743973000	-0.849729000	-0.615609000
O	-0.082748000	0.513317000	-0.945519000
C	0.242090000	2.835479000	-0.382474000
H	0.632955000	2.617889000	0.620805000
H	1.094817000	2.922684000	-1.073706000
H	-0.300972000	3.787744000	-0.371311000
C	-3.756585000	-1.608182000	-0.258763000
H	-3.419424000	-2.651193000	-0.198044000

H	-4.590135000	-1.479120000	0.445948000
H	-4.133338000	-1.430222000	-1.276426000
Al	1.142760000	-0.397073000	0.002945000
Cl	0.828656000	-2.450163000	-0.529855000
Cl	3.089993000	0.323082000	-0.535648000
Cl	0.705514000	-0.046043000	2.089683000

TS (*Z*)-hex-4-en-2-ol: $AlCl_3$
***E* =** -1933.331483
***G* =** -1933.373933
N_{imag} = -252.2936 cm^{-1}
***SP* =** -1934.409369

C	-0.921995000	1.647652000	-0.777523000
C	-2.401343000	1.279084000	0.319841000
C	-3.030730000	0.392489000	-0.535258000
C	-2.714997000	-1.034098000	-0.649634000
H	-1.883368000	0.893991000	1.203664000
H	-2.843926000	2.273113000	0.417913000
H	-3.702670000	0.815594000	-1.292035000
H	-1.409171000	1.969478000	-1.709884000
H	-1.770503000	-0.997234000	-1.250314000
O	-0.201574000	0.564561000	-0.921200000
C	-0.303151000	2.802882000	-0.016280000
H	0.003395000	2.504776000	0.995064000
H	0.597144000	3.094313000	-0.579396000
H	-0.980116000	3.663758000	0.036466000
C	-2.443052000	-1.805555000	0.639134000
H	-3.344454000	-1.844317000	1.267467000
H	-2.146617000	-2.833853000	0.395262000
H	-1.628730000	-1.360380000	1.225885000
H	-3.456108000	-1.536190000	-1.286410000
Al	1.125613000	-0.245771000	-0.033931000
Cl	1.055291000	-2.290258000	-0.683165000
Cl	2.963720000	0.735913000	-0.547399000
Cl	0.712539000	-0.072668000	2.083820000

(*E*)-hex-4-en-2-ol - $AlCl_3$
***E* =** -1933.371642
***G* =** -1933.415396
N_{imag} = 0
***SP* =** -1934.449282

C	-0.597106000	1.909161000	-0.744361000
C	-1.794494000	1.845015000	0.204296000
C	-2.793932000	0.815987000	-0.247602000
C	-2.877848000	-0.414245000	0.276000000
H	-1.427032000	1.608427000	1.214963000
H	-2.246013000	2.848145000	0.237769000
H	-3.440929000	1.086404000	-1.092036000
H	-2.223527000	-0.661862000	1.123355000
H	-0.924104000	2.208954000	-1.751098000
H	-0.837326000	-0.026063000	-1.178024000
O	-0.085773000	0.542435000	-0.918269000
C	0.532147000	2.798705000	-0.281454000
H	0.877717000	2.515642000	0.723827000
H	1.377881000	2.759582000	-0.980999000
H	0.167209000	3.833706000	-0.235708000
C	-3.801078000	-1.497128000	-0.182429000
H	-3.226183000	-2.391752000	-0.469506000
H	-4.476628000	-1.803756000	0.631643000

H	-4.410144000	-1.180538000	-1.041158000
Al	1.161951000	-0.490181000	0.052163000
Cl	0.440603000	-2.409613000	-0.552603000
Cl	3.088058000	0.046355000	-0.667050000
Cl	0.845679000	-0.078447000	2.121905000

(*Z*)-hex-4-en-2-ol - $AlCl_3$
***E* =** -1933.370765
***G* =** -1933.413988
N_{imag} = 0
***SP* =** -1934.448858

C	-1.021557000	1.755714000	-0.589029000
C	-2.328216000	1.399878000	0.116402000
C	-3.101586000	0.368763000	-0.659909000
C	-3.145462000	-0.948978000	-0.410227000
H	-2.094428000	1.057627000	1.135865000
H	-2.914816000	2.326347000	0.208974000
H	-3.637477000	0.740848000	-1.540233000
H	-1.226306000	2.142393000	-1.598439000
H	-0.890117000	-0.139417000	-1.227800000
O	-0.283976000	0.504176000	-0.811557000
C	-0.143478000	2.724350000	0.166842000
H	0.077469000	2.354532000	1.178508000
H	0.799391000	2.906194000	-0.366338000
H	-0.675918000	3.680432000	0.259181000
C	-2.501434000	-1.692278000	0.719836000
H	-3.267453000	-2.206948000	1.320911000
H	-1.829733000	-2.473224000	0.329731000
H	-1.912780000	-1.050249000	1.388443000
H	-3.735359000	-1.564995000	-1.098678000
Al	1.207368000	-0.310202000	0.003193000
Cl	0.987941000	-2.248341000	-0.865886000
Cl	2.925247000	0.770553000	-0.633223000
Cl	0.871161000	-0.252742000	2.110716000

Acetaldehyde-BF_3
***E* =** -477.849351
***G* =** -477.881698
N_{imag} = 0
***SP* =** -478.490012

C	1.536310000	0.802903000	0.009437000
H	2.191241000	1.690785000	-0.000771000
O	0.327051000	1.032977000	0.010222000
B	-0.863239000	-0.092225000	-0.000814000
F	-1.992819000	0.662633000	-0.042132000
F	-0.635141000	-0.830930000	-1.129162000
F	-0.695550000	-0.796054000	1.160006000
C	2.177825000	-0.522441000	0.003187000
H	1.473880000	-1.346705000	0.145460000
H	2.694722000	-0.622205000	-0.966477000
H	2.966711000	-0.528181000	0.769921000

TS (*E*)-hex-4-en-2-ol: BF_3
***E* =** -634.785821
***G* =** -634.824755
N_{imag} = -285.6531 cm^{-1}
***SP* =** -635.705473

C	0.422599000	1.290045000	-0.681820000
C	-0.850775000	1.662028000	0.635213000

C	-1.959506000	1.024463000	0.103180000
C	-2.169797000	-0.415374000	0.185257000
H	-0.344489000	1.176826000	1.473930000
H	-0.857098000	2.754726000	0.629609000
H	-2.622433000	1.602685000	-0.554663000
H	-1.842650000	-0.803270000	1.162147000
H	-0.027539000	1.783429000	-1.554492000
H	-1.352151000	-0.769194000	-0.501000000
O	0.536137000	-0.003875000	-0.865887000
B	1.308684000	-0.933023000	0.041233000
F	0.897775000	-2.209686000	-0.300931000
F	2.669017000	-0.757848000	-0.170201000
F	0.977283000	-0.642257000	1.372878000
C	1.612974000	2.051223000	-0.132502000
H	1.945357000	1.647300000	0.830772000
H	2.428881000	1.927527000	-0.859675000
H	1.386253000	3.119391000	-0.033655000
C	-3.526142000	-0.945204000	-0.256219000
H	-3.513008000	-2.042506000	-0.288377000
H	-4.317492000	-0.636393000	0.441498000
H	-3.788951000	-0.579371000	-1.259519000

TS (*Z*)-hex-4-en-2-ol: BF_3

***E* =** -634.785500

***G* =** -634.823215

N_{imag} = -170.6826 cm^{-1}

***SP* =** -635.704856

C	0.197223000	1.426044000	-0.525849000
C	-1.224764000	1.443510000	0.565101000
C	-2.183885000	0.897773000	-0.289028000
C	-2.330905000	-0.524178000	-0.555095000
H	-0.856743000	0.821782000	1.386062000
H	-1.341330000	2.507028000	0.789722000
H	-2.726968000	1.589517000	-0.945064000
H	-0.142637000	2.013220000	-1.391587000
H	-1.410458000	-0.655058000	-1.195001000
O	0.497825000	0.187814000	-0.891244000
B	1.283661000	-0.773736000	-0.051549000
F	0.982348000	-2.037115000	-0.536035000
F	2.639773000	-0.492311000	-0.170424000
F	0.888471000	-0.657705000	1.296373000
C	1.228498000	2.196558000	0.284834000
H	1.447911000	1.699375000	1.236599000
H	2.150249000	2.225208000	-0.313527000
H	0.891243000	3.224184000	0.467866000
C	-2.222286000	-1.502587000	0.612276000
H	-3.052627000	-1.358481000	1.318185000
H	-2.271127000	-2.529903000	0.228886000
H	-1.270643000	-1.390401000	1.144852000
H	-3.196393000	-0.718838000	-1.201963000

(*E*)-hex-4-en-2-ol - BF_3

***E* =** -634.828828

***G* =** -634.868156

N_{imag} = 0

***SP* =** -635.75017

C	0.418784000	1.485539000	-0.505287000
C	-0.742514000	1.638494000	0.478245000
C	-1.991473000	0.984111000	-0.044170000

C	-2.413722000	-0.227730000	0.339822000
H	-0.447235000	1.196176000	1.440519000
H	-0.902172000	2.716259000	0.634130000
H	-2.545807000	1.522141000	-0.824148000
H	-1.845351000	-0.750967000	1.120405000
H	0.161232000	1.960224000	-1.463899000
H	-0.316319000	-0.288261000	-1.054423000
O	0.567577000	0.067837000	-0.843627000
B	1.328551000	-1.032643000	0.062212000
F	0.725726000	-2.190909000	-0.355782000
F	2.648754000	-0.931757000	-0.263755000
F	1.066612000	-0.729684000	1.375240000
C	1.740741000	2.024485000	-0.009061000
H	2.018167000	1.570418000	0.951196000
H	2.538983000	1.839727000	-0.739453000
H	1.640991000	3.109068000	0.132982000
C	-3.619570000	-0.935535000	-0.191530000
H	-3.342798000	-1.915993000	-0.609992000
H	-4.342537000	-1.131441000	0.616233000
H	-4.123816000	-0.351865000	-0.975032000

(*Z*)-hex-4-en-2-ol - BF_3
***E* =** -634.828203
***G* =** -634.868140
N_{imag} = 0
***SP* =** -635.75029

C	-0.050113000	1.292006000	-0.245559000
C	-1.571747000	1.390990000	-0.142604000
C	-2.244167000	0.296921000	-0.926241000
C	-2.767666000	-0.834627000	-0.428884000
H	-1.859837000	1.370022000	0.918985000
H	-1.861796000	2.375207000	-0.539362000
H	-2.250445000	0.427116000	-2.014117000
H	0.268700000	1.424328000	-1.290547000
H	-0.354813000	-0.685279000	-0.254124000
O	0.305411000	-0.074185000	0.127954000
B	1.789624000	-0.663284000	-0.058472000
F	1.547780000	-1.996116000	-0.271987000
F	2.323840000	-0.016735000	-1.143082000
F	2.446337000	-0.392996000	1.108145000
C	0.695737000	2.242653000	0.661095000
H	0.451733000	2.045691000	1.714693000
H	1.780246000	2.149488000	0.522315000
H	0.404259000	3.273430000	0.417069000
C	-2.877241000	-1.245807000	1.008107000
H	-3.935267000	-1.387719000	1.279039000
H	-2.381641000	-2.216415000	1.167510000
H	-2.437783000	-0.520153000	1.704838000
H	-3.185201000	-1.546014000	-1.150751000

Acetaldehyde-$TiCl_4$
***E* =** -2843.459219
***G* =** -2843.500010
N_{imag} = 0
***SP* =** -2844.448333

C	2.725056000	-0.077024000	0.696025000
H	3.441346000	-0.093559000	1.540978000
O	1.535704000	-0.098836000	0.971683000
C	3.284832000	0.011011000	-0.671856000
H	2.578897000	-0.345618000	-1.431291000

H	3.499075000	1.078612000	-0.856689000
H	4.239863000	-0.527039000	-0.729675000
Cl	-1.244485000	-0.040350000	2.050583000
Cl	0.308570000	2.002649000	-0.615437000
Cl	0.161564000	-2.001316000	-0.750186000
Cl	-2.324635000	0.098877000	-1.095867000
Ti	-0.428244000	0.002580000	0.024709000

TS (*E*)-hex-4-en-2-ol: $TiCl_4$
***E* =** -3000.394503
***G* =** -3000.440874
N_{imag} = -354.9976 cm^{-1}
***SP* =** -3001.662255

C	-1.328621000	1.744878000	-0.598740000
C	-2.803953000	1.370123000	0.620100000
C	-3.351448000	0.291650000	-0.038082000
C	-2.832563000	-1.075903000	0.057618000
H	-2.194943000	1.182528000	1.509502000
H	-3.333714000	2.323813000	0.568528000
H	-4.130608000	0.483932000	-0.788125000
H	-2.397391000	-1.257937000	1.052216000
H	-1.904035000	1.916095000	-1.520659000
H	-1.938443000	-1.029537000	-0.610513000
O	-0.545079000	0.714435000	-0.645126000
C	-0.778725000	2.995603000	0.039507000
H	-0.369688000	2.781859000	1.035504000
H	0.050968000	3.321251000	-0.608019000
H	-1.525701000	3.795905000	0.092437000
C	-3.765497000	-2.190233000	-0.397758000
H	-3.226069000	-3.146359000	-0.418021000
H	-4.620052000	-2.296623000	0.285435000
H	-4.154206000	-1.998497000	-1.408675000
Cl	0.573129000	-2.051069000	-1.086741000
Cl	2.172306000	1.322793000	-1.325065000
Cl	0.160225000	0.007470000	2.061972000
Cl	2.990975000	-0.875597000	0.870275000
Ti	1.046662000	-0.157717000	-0.044181000

TS (*Z*)-hex-4-en-2-ol: $TiCl_4$
***E* =** -3000.394483
***G* =** -3000.440346
N_{imag} = -326.8064 cm^{-1}
***SP* =** -3001.662414

C	-1.617591000	1.504112000	-0.573009000
C	-3.054858000	0.812897000	0.534242000
C	-3.474043000	-0.102775000	-0.404473000
C	-2.903285000	-1.444424000	-0.609313000
H	-2.455759000	0.489129000	1.390249000
H	-3.655180000	1.715008000	0.668125000
H	-4.204659000	0.239363000	-1.147446000
H	-2.134189000	1.668436000	-1.530755000
H	-2.016710000	-1.248199000	-1.257105000
O	-0.684723000	0.601978000	-0.625099000
C	-1.310631000	2.778102000	0.176121000
H	-0.976749000	2.568885000	1.199851000
H	-0.473356000	3.243523000	-0.368442000
H	-2.161770000	3.468679000	0.177383000
C	-2.450422000	-2.208743000	0.630003000
H	-3.299568000	-2.414053000	1.298159000
H	-2.009654000	-3.168570000	0.329577000

H	-1.687888000	-1.658236000	1.196709000
H	-3.588434000	-2.038477000	-1.231422000
Cl	0.769675000	-1.852447000	-1.379074000
Cl	1.968753000	1.689819000	-1.067633000
Cl	0.208570000	0.099234000	2.089871000
Cl	2.988013000	-0.785437000	0.772846000
Ti	1.005069000	-0.058463000	-0.059895000

(*E*)-hex-4-en-2-ol - $TiCl_4$

***E* =** -3000.438039

***G* =** -3000.483852

N_{imag} = 0

***SP* =** -3001.707015

C	-1.538309000	1.853006000	-0.510762000
C	-2.633033000	1.421481000	0.471282000
C	-3.372253000	0.211794000	-0.028435000
C	-3.117358000	-1.039109000	0.375340000
H	-2.165187000	1.212800000	1.445732000
H	-3.320147000	2.271214000	0.604823000
H	-4.117409000	0.376673000	-0.817717000
H	-2.364070000	-1.186713000	1.161121000
H	-1.998758000	2.105884000	-1.480513000
H	-1.276118000	-0.033217000	-1.004325000
O	-0.688072000	0.708246000	-0.774332000
C	-0.713385000	3.026684000	-0.034011000
H	-0.224126000	2.808810000	0.925505000
H	0.053831000	3.294181000	-0.771627000
H	-1.378396000	3.890391000	0.105296000
C	-3.773485000	-2.275643000	-0.151434000
H	-3.018156000	-2.965449000	-0.560438000
H	-4.292458000	-2.817676000	0.655109000
H	-4.502020000	-2.046399000	-0.942327000
Cl	0.153458000	-1.980770000	-1.002186000
Cl	2.212507000	1.422727000	-1.085845000
Cl	0.539630000	0.294913000	2.009674000
Cl	2.959654000	-1.342173000	0.497703000
Ti	1.149331000	-0.203437000	-0.044852000

(*Z*)-hex-4-en-2-ol - $TiCl_4$

***E* =** -3000.438088

***G* =** -3000.484568

N_{imag} = 0

***SP* =** -3001.708144

C	-1.662918000	1.515135000	-0.261369000
C	-3.137124000	1.113920000	-0.121419000
C	-3.452752000	-0.119373000	-0.922005000
C	-3.512026000	-1.376569000	-0.454079000
H	-3.362606000	0.968979000	0.945909000
H	-3.746681000	1.960525000	-0.470895000
H	-3.585630000	0.029590000	-1.999173000
H	-1.454000000	1.808163000	-1.304360000
H	-1.375463000	-0.433504000	-0.317447000
O	-0.871421000	0.336659000	0.008764000
C	-1.257523000	2.627752000	0.678721000
H	-1.421502000	2.333948000	1.725458000
H	-0.200316000	2.892570000	0.544446000
H	-1.863551000	3.519124000	0.463737000
C	-3.351354000	-1.841468000	0.962245000
H	-4.263158000	-2.359376000	1.298736000
H	-2.529897000	-2.572062000	1.029511000

H	-3.136736000	-1.027074000	1.666533000
H	-3.711526000	-2.169004000	-1.184574000
Cl	0.339331000	-2.181730000	-0.354511000
Cl	1.393705000	1.488412000	-1.517402000
Cl	1.362978000	0.589043000	2.036286000
Cl	3.310237000	-0.843904000	-0.234799000
Ti	1.225379000	-0.138296000	-0.025605000

Acetaldehyde-FeCl$_3$
***E* =** -2797.518102
***G* =** -2797.558965
N_{imag} = 0
***SP* =** -2798.411696

C	3.771463000	0.000454000	-0.679441000
C	2.436561000	-0.000423000	-0.057015000
O	1.394893000	0.000426000	-0.707740000
H	2.373112000	-0.001971000	1.048881000
H	4.322863000	0.880127000	-0.310396000
H	4.322801000	-0.880417000	-0.313148000
H	3.713063000	0.002177000	-1.773343000
Fe	-0.526075000	-0.000002000	-0.002297000
Cl	-1.392883000	1.867112000	-0.747740000
Cl	-1.392807000	-1.866690000	-0.748874000
Cl	-0.123790000	-0.000625000	2.172402000

TS (*E*)-hex-4-en-2-ol: FeCl$_3$
***E* =** -2954.455291
***G* =** -2954.500699
N_{imag} = -320.6109 cm^{-1}
***SP* =** -2955.626654

C	-0.794597000	1.778530000	-0.949010000
C	-2.173735000	1.761647000	0.328906000
C	-2.962110000	0.727173000	-0.145286000
C	-2.650341000	-0.680212000	0.074196000
H	-1.565253000	1.561413000	1.217363000
H	-2.564484000	2.776759000	0.222016000
H	-3.762125000	0.967200000	-0.858489000
H	-2.163567000	-0.829522000	1.050563000
H	-1.368701000	2.050211000	-1.849385000
H	-1.800196000	-0.801468000	-0.650668000
O	-0.231485000	0.603875000	-1.034413000
C	0.055039000	2.914383000	-0.415369000
H	0.468033000	2.668597000	0.572783000
H	0.892728000	3.034439000	-1.119443000
H	-0.503549000	3.856595000	-0.364222000
C	-3.745005000	-1.696976000	-0.213544000
H	-3.328271000	-2.712015000	-0.180531000
H	-4.547741000	-1.633748000	0.534580000
H	-4.186406000	-1.540715000	-1.208664000
Cl	0.663715000	-2.483571000	-0.506418000
Cl	3.060769000	0.388103000	-0.526301000
Cl	0.549237000	0.111197000	2.136330000
Fe	1.048179000	-0.358593000	0.002640000

TS (*Z*)-hex-4-en-2-ol: FeCl$_3$
***E* =** -2954.455251
***G* =** -2954.500185
N_{imag} = -269.4233 cm^{-1}
***SP* =** -2955.626489

C	-1.128018000	1.704994000	-0.816409000

C	-2.556045000	1.221308000	0.249921000
C	-3.126638000	0.266030000	-0.579844000
C	-2.693199000	-1.127417000	-0.659285000
H	-2.023359000	0.886629000	1.146282000
H	-3.086283000	2.172923000	0.338245000
H	-3.826544000	0.620573000	-1.346489000
H	-1.633093000	2.032528000	-1.739633000
H	-1.703930000	-0.993823000	-1.174956000
O	-0.336146000	0.677540000	-1.006173000
C	-0.557414000	2.870882000	-0.028599000
H	-0.210452000	2.549231000	0.962501000
H	0.312611000	3.232077000	-0.597796000
H	-1.281160000	3.688792000	0.072315000
C	-2.452203000	-1.879323000	0.649471000
H	-3.398882000	-2.032512000	1.186601000
H	-2.012758000	-2.860171000	0.427754000
H	-1.751639000	-1.351873000	1.309917000
H	-3.330234000	-1.694414000	-1.350669000
Fe	1.026301000	-0.184832000	-0.006049000
Cl	0.938970000	-2.316609000	-0.634583000
Cl	2.941464000	0.763127000	-0.585375000
Cl	0.533223000	0.070923000	2.165903000

(*E*)-hex-4-en-2-ol - $FeCl_3$
***E* =** -2954.498439
***G* =** -2954.543639
N_{imag} = 0
***SP* =** -2955.671198

C	-0.851048000	1.995899000	-0.743506000
C	-1.964962000	1.806940000	0.287619000
C	-2.954325000	0.764822000	-0.155487000
C	-2.934576000	-0.506448000	0.265713000
H	-1.502344000	1.515597000	1.243645000
H	-2.456579000	2.780767000	0.435081000
H	-3.693693000	1.069035000	-0.907611000
H	-2.186046000	-0.793191000	1.016607000
H	-1.279316000	2.324593000	-1.703934000
H	-0.967560000	0.094550000	-1.292043000
O	-0.251604000	0.698645000	-1.022486000
C	0.235835000	2.957694000	-0.320097000
H	0.647383000	2.692394000	0.665525000
H	1.051488000	2.971357000	-1.055734000
H	-0.188107000	3.968481000	-0.249169000
C	-3.859079000	-1.589298000	-0.191690000
H	-3.286488000	-2.426175000	-0.621849000
H	-4.427289000	-2.000205000	0.657859000
H	-4.572781000	-1.228254000	-0.946140000
Cl	0.494879000	-2.359858000	-0.819696000
Cl	3.066504000	0.357255000	-0.479826000
Cl	0.586623000	-0.239809000	2.182287000
Fe	1.089500000	-0.423644000	0.041359000

(*Z*)-hex-4-en-2-ol - $FeCl_3$
***E* =** -2954.495086
***G* =** -2954.541157
N_{imag} = 0
***SP* =** -2955.669551

C	1.228344000	-1.563542000	-0.182795000
C	2.736953000	-1.413230000	0.030548000
C	3.296837000	-0.290025000	-0.798561000

C	3.524056000	0.967908000	-0.387965000
H	2.925618000	-1.259951000	1.103921000
H	3.209519000	-2.365781000	-0.251023000
H	3.476833000	-0.519758000	-1.854466000
H	1.019221000	-1.825271000	-1.233355000
H	1.228778000	0.415192000	-0.331933000
O	0.634091000	-0.259688000	0.049965000
C	0.582036000	-2.565285000	0.746644000
H	0.680887000	-2.241267000	1.792657000
H	-0.482654000	-2.702872000	0.513411000
H	1.078912000	-3.538226000	0.629749000
C	3.342041000	1.536016000	0.987900000
H	4.284576000	1.978777000	1.345360000
H	2.600174000	2.350642000	0.968422000
H	3.007957000	0.793348000	1.724151000
H	3.894561000	1.675279000	-1.138728000
Fe	-1.304974000	0.307781000	-0.056883000
Cl	-0.877713000	2.441976000	-0.401422000
Cl	-2.002495000	-0.942615000	-1.737197000
Cl	-2.197991000	-0.247359000	1.870175000

Acetaldehyde-FeBr$_3$
***E* =** -9139.068384
***G* =** -9139.112566
N_{imag} = 0
***SP* =** -9140.426099

C	1.782276000	0.172659000	2.485156000
H	1.755320000	0.293721000	3.584820000
O	0.701109000	0.145911000	1.907780000
C	3.105092000	0.058777000	1.844843000
H	3.035446000	-0.059809000	0.757468000
H	3.691439000	0.954164000	2.107015000
H	3.632602000	-0.795581000	2.298470000
Fe	-0.098800000	0.000805000	0.050048000
Br	1.432545000	-0.641725000	-1.600223000
Br	-0.941129000	2.150361000	-0.267628000
Br	-1.762248000	-1.593474000	0.402387000

TS (*E*)-hex-4-en-2-ol: FeBr$_3$
***E* =** -9296.007046
***G* =** -9296.056149
N_{imag} = -326.0787 cm^{-1}
***SP* =** -9297.644089

C	1.178371000	-1.807172000	-1.380288000
C	2.680853000	-2.000305000	-0.234567000
C	3.437339000	-0.915231000	-0.635664000
C	3.189296000	0.448367000	-0.176007000
H	2.157405000	-1.928286000	0.724760000
H	3.027897000	-2.994042000	-0.528075000
H	4.151326000	-1.055910000	-1.458346000
H	2.880472000	0.454525000	0.881590000
H	1.671163000	-1.925678000	-2.358065000
H	2.231826000	0.669498000	-0.718514000
O	0.638291000	-0.633834000	-1.211922000
C	0.369010000	-3.018758000	-0.965973000
H	0.053736000	-2.954415000	0.084261000
H	-0.533882000	-3.019110000	-1.596508000
H	0.917917000	-3.952816000	-1.135481000
C	4.243705000	1.495759000	-0.500690000
H	3.878612000	2.493904000	-0.225472000

H	5.174260000	1.308184000	0.053431000
H	4.477642000	1.504789000	-1.575269000
Fe	-0.624559000	0.165820000	-0.036374000
Br	-0.386437000	2.493562000	-0.309876000
Br	-2.757789000	-0.629726000	-0.643992000
Br	0.014295000	-0.522615000	2.149638000

TS (*Z*)-hex-4-en-2-ol: $FeBr_3$
***E* =** -9296.006103
***G* =** -9296.053626
N_{imag} = -278.5350 cm^{-1}
***SP* =** -9297.644264

C	-2.967718000	-1.594643000	-0.276507000
C	-3.571149000	-0.584249000	-1.009961000
C	-3.294870000	0.843298000	-0.854897000
C	-1.421297000	-1.751887000	-1.284696000
H	-2.544009000	-1.356527000	0.704618000
H	-3.392173000	-2.596396000	-0.380777000
H	-1.822944000	-1.945071000	-2.292383000
H	-2.304475000	0.912079000	-1.377562000
O	-0.727898000	-0.641893000	-1.214035000
Fe	0.579523000	0.107875000	-0.080047000
C	-0.802030000	-2.990604000	-0.665734000
H	0.142077000	-3.167957000	-1.203573000
H	-0.567917000	-2.832548000	0.395516000
H	-1.446445000	-3.870199000	-0.781740000
H	-4.168282000	-0.884699000	-1.879921000
H	-3.996209000	1.435451000	-1.458289000
C	-3.134868000	1.392613000	0.560066000
H	-2.894262000	2.462565000	0.510055000
H	-4.065723000	1.272955000	1.132658000
H	-2.319075000	0.898930000	1.105043000
Br	0.030986000	-0.457152000	2.162676000
Br	2.658061000	-0.840968000	-0.654668000
Br	0.490571000	2.444253000	-0.407716000

(*E*)-hex-4-en-2-ol - $FeBr_3$
***E* =** -9296.049354
***G* =** -9296.098347
N_{imag} = 0
***SP* =** -9297.688093

C	1.241460000	-2.005036000	-1.369293000
C	2.391002000	-2.078786000	-0.363363000
C	3.399514000	-0.987188000	-0.590083000
C	3.420364000	0.162083000	0.097061000
H	1.967109000	-2.007136000	0.651055000
H	2.857924000	-3.070898000	-0.459875000
H	4.120383000	-1.137437000	-1.404096000
H	2.690892000	0.296577000	0.907676000
H	1.632532000	-2.105473000	-2.394355000
H	1.378406000	-0.030940000	-1.472209000
O	0.659449000	-0.670311000	-1.316131000
C	0.151142000	-3.025642000	-1.142011000
H	-0.237545000	-2.978560000	-0.112758000
H	-0.678299000	-2.875570000	-1.846616000
H	0.564428000	-4.030905000	-1.301351000
C	4.371955000	1.293436000	-0.130457000
H	3.823667000	2.220945000	-0.359926000
H	4.961346000	1.493676000	0.778397000
H	5.066397000	1.083633000	-0.956644000

Fe	-0.639254000	0.194022000	0.002648000
Br	0.047516000	2.407498000	-0.323969000
Br	-2.803827000	-0.255247000	-0.720189000
Br	-0.290971000	-0.629199000	2.170437000

(*Z*)-hex-4-en-2-ol - $FeBr_3$

***E* =** -9296.048711

***G* =** -9296.097699

N_{imag} = 0

***SP* =** -9297.687438

C	-2.854312000	-1.695684000	-0.474937000
C	-3.622919000	-0.554532000	-1.083080000
C	-3.749225000	0.684443000	-0.583873000
C	-1.524616000	-1.904535000	-1.199137000
H	-2.651903000	-1.531482000	0.594443000
H	-3.430750000	-2.629831000	-0.559331000
H	-1.711031000	-2.114891000	-2.264567000
H	-1.408065000	0.054124000	-1.481556000
O	-0.796007000	-0.643513000	-1.180596000
Fe	0.644592000	0.146801000	0.015004000
C	-0.653791000	-2.989565000	-0.611875000
H	0.298873000	-3.065272000	-1.154280000
H	-0.444843000	-2.797984000	0.451247000
H	-1.176286000	-3.952328000	-0.693239000
H	-4.081988000	-0.759653000	-2.057127000
H	-4.322406000	1.402662000	-1.181756000
C	-3.229779000	1.209426000	0.718818000
H	-2.621406000	2.112090000	0.553213000
H	-4.068271000	1.508603000	1.367995000
H	-2.611325000	0.486518000	1.266669000
Br	0.201561000	-0.413947000	2.244555000
Br	2.662692000	-0.731443000	-0.769905000
Br	0.325630000	2.406001000	-0.514151000

Acetaldehyde-$InCl_3$

***E* =** -1724.153330

***G* =** -1724.194258

N_{imag} = 0

***SP* =** -1724.871989

C	-2.446621000	0.104347000	-1.457982000
H	-2.924136000	0.242987000	-2.446986000
O	-1.221105000	0.126480000	-1.432022000
C	-3.336646000	-0.071475000	-0.294858000
H	-2.790746000	-0.329085000	0.620417000
H	-3.890453000	0.872937000	-0.160363000
H	-4.089624000	-0.836884000	-0.535273000
Cl	0.789431000	2.295067000	0.443744000
Cl	-0.533111000	-1.174161000	1.915058000
Cl	2.000113000	-1.185713000	-1.172985000
In	0.404164000	-0.001170000	0.088501000

TS (*E*)-hex-4-en-2-ol: $InCl_3$

***E* =** -1881.088047

***G* =** -1881.133674

N_{imag} = -276.7610 cm^{-1}

***SP* =** -1882.085605

C	-1.139552000	1.796808000	-0.980921000
C	-2.434263000	1.735169000	0.303062000
C	-3.229120000	0.675068000	-0.127293000

C	-2.840072000	-0.707081000	0.061203000
H	-1.815637000	1.550420000	1.188781000
H	-2.873617000	2.733947000	0.233756000
H	-4.063463000	0.889701000	-0.808587000
H	-2.338194000	-0.855968000	1.030405000
H	-1.728534000	2.099149000	-1.862516000
H	-1.971691000	-0.694003000	-0.670088000
O	-0.565497000	0.624177000	-1.144483000
C	-0.262994000	2.924552000	-0.463965000
H	0.180559000	2.674450000	0.510938000
H	0.548696000	3.064874000	-1.193442000
H	-0.819920000	3.865282000	-0.371101000
C	-3.830026000	-1.804494000	-0.288856000
H	-3.317087000	-2.774593000	-0.314845000
H	-4.632186000	-1.863044000	0.460019000
H	-4.286665000	-1.632475000	-1.274084000
Cl	0.587061000	-2.560744000	-0.465384000
Cl	3.031054000	0.613927000	-0.661852000
Cl	0.370672000	0.260826000	2.289627000
In	0.943847000	-0.267540000	0.029523000

TS (*Z*)-hex-4-en-2-ol: $InCl_3$
***E* =** -1881.087728
***G* =** -1881.132452
N_{imag} = -161.9280 cm^{-1}
***SP* =** -1882.085046

C	1.448327000	-1.660848000	-0.829099000
C	2.761459000	-1.236608000	0.246055000
C	3.425983000	-0.265266000	-0.520758000
C	2.982543000	1.103317000	-0.636602000
H	2.234911000	-0.892040000	1.143544000
H	3.302537000	-2.178524000	0.375637000
H	4.165420000	-0.620164000	-1.249356000
H	1.959412000	-2.038846000	-1.731153000
H	2.037018000	0.834771000	-1.219694000
O	0.695266000	-0.599305000	-1.109152000
C	0.758610000	-2.801682000	-0.091179000
H	0.382714000	-2.475815000	0.889589000
H	-0.094060000	-3.124860000	-0.706555000
H	1.433769000	-3.655148000	0.051370000
C	2.552634000	1.874915000	0.607552000
H	3.433688000	2.159089000	1.199953000
H	2.024083000	2.788533000	0.308285000
H	1.876127000	1.295788000	1.249845000
H	3.621962000	1.690962000	-1.306567000
Cl	-0.971390000	2.427009000	-0.699546000
Cl	-2.838652000	-1.099026000	-0.599671000
Cl	-0.344671000	-0.081453000	2.312317000
In	-0.916060000	0.157892000	-0.000208000

(*E*)-hex-4-en-2-ol - $InCl_3$
***E* =** -1881.134903
***G* =** -1881.181830
N_{imag} = 0
***SP* =** -1882.134377

C	-1.092446000	2.053576000	-0.850884000
C	-2.208528000	1.843422000	0.175936000
C	-3.144298000	0.744376000	-0.242948000
C	-3.096156000	-0.495962000	0.259500000
H	-1.746468000	1.599289000	1.145620000

H	-2.742494000	2.799151000	0.291824000
H	-3.858101000	0.972904000	-1.045024000
H	-2.377781000	-0.698709000	1.065953000
H	-1.516289000	2.406310000	-1.804338000
H	-1.185727000	0.149201000	-1.409076000
O	-0.478940000	0.769544000	-1.153453000
C	-0.002987000	2.996122000	-0.392337000
H	0.417468000	2.674483000	0.573641000
H	0.803361000	3.056114000	-1.136097000
H	-0.424796000	4.000774000	-0.253958000
C	-3.946825000	-1.648475000	-0.168408000
H	-3.311966000	-2.481059000	-0.510409000
H	-4.541387000	-2.029998000	0.676618000
H	-4.632986000	-1.371457000	-0.981462000
Cl	0.124518000	-2.451966000	-0.634828000
Cl	3.051577000	0.352800000	-0.749636000
Cl	0.487742000	0.195630000	2.324901000
In	0.971651000	-0.363910000	0.080444000

(*Z*)-hex-4-en-2-ol - $InCl_3$
***E* =** -1881.134369
***G* =** -1881.179945
N_{imag} = 0
***SP* =** -1882.133959

C	-1.499527000	1.811660000	-0.715170000
C	-2.692092000	1.352338000	0.123460000
C	-3.494259000	0.309590000	-0.605435000
C	-3.457787000	-1.015896000	-0.402897000
H	-2.314094000	0.980906000	1.088476000
H	-3.315143000	2.235269000	0.333665000
H	-4.130697000	0.683582000	-1.415566000
H	-1.850563000	2.221227000	-1.675849000
H	-1.284848000	-0.027126000	-1.447144000
O	-0.697712000	0.646348000	-1.059209000
C	-0.607567000	2.818918000	-0.026171000
H	-0.277183000	2.449372000	0.957195000
H	0.273302000	3.051717000	-0.640688000
H	-1.169038000	3.748522000	0.137200000
C	-2.677087000	-1.768076000	0.630964000
H	-3.354538000	-2.372915000	1.253936000
H	-1.980699000	-2.473379000	0.149663000
H	-2.091398000	-1.119906000	1.296517000
H	-4.083606000	-1.635044000	-1.056217000
Cl	0.816244000	-2.376160000	-0.924866000
Cl	2.821388000	1.099400000	-0.631954000
Cl	0.397396000	-0.048969000	2.326971000
In	1.002748000	-0.233200000	0.048396000

Acetaldehyde-FeF_3
***E* =** -1716.521047
***G* =** -1716.560138
N_{imag} = 0
***SP* =** -1717.327071

C	2.136396000	-0.848350000	-0.003810000
H	2.838525000	-1.703202000	0.031564000
O	0.939383000	-1.115652000	-0.007305000
C	2.703532000	0.514151000	-0.024754000
H	1.935145000	1.267608000	-0.237413000
H	3.161872000	0.693324000	0.962740000
H	3.522101000	0.554309000	-0.758120000

Fe	-0.741046000	0.065274000	-0.001858000
F	-0.218791000	1.560926000	-0.851674000
F	-1.032377000	0.311839000	1.735865000
F	-1.942729000	-0.937072000	-0.853151000

TS (*E*)-hex-4-en-2-ol: FeF_3

***E* =** -1873.455089

***G* =** -1873.498075

N_{imag} = -278.5587 cm^{-1}

***SP* =** -1874.538748

C	-0.335405000	1.671991000	-0.743316000
C	-1.511975000	1.615006000	0.655415000
C	-2.492565000	0.755133000	0.171972000
C	-2.313681000	-0.686040000	0.121115000
H	-0.834022000	1.196297000	1.407711000
H	-1.794359000	2.664281000	0.773137000
H	-3.358537000	1.185918000	-0.348855000
H	-1.747739000	-1.041468000	0.996230000
H	-0.952769000	2.114948000	-1.542714000
H	-1.530301000	-0.722098000	-0.691692000
O	0.097980000	0.468211000	-1.039745000
C	0.703234000	2.645401000	-0.209345000
H	1.211376000	2.238671000	0.675822000
H	1.454013000	2.780755000	-1.001905000
H	0.263801000	3.620886000	0.032964000
C	-3.519501000	-1.539530000	-0.236510000
H	-3.211000000	-2.578905000	-0.408866000
H	-4.259201000	-1.537052000	0.576505000
H	-4.010026000	-1.173785000	-1.150170000
Fe	1.291758000	-0.618459000	0.026450000
F	0.516173000	-0.426497000	1.670305000
F	2.922496000	0.136242000	0.033878000
F	1.141172000	-2.285965000	-0.620171000

TS (*Z*)-hex-4-en-2-ol: FeF_3

***E* =** -1873.456266

***G* =** -1873.498993

N_{imag} = -108.2823 cm^{-1}

***SP* =** -1874.537536

C	-0.775393000	1.608307000	-0.563625000
C	-1.970387000	1.135708000	0.546827000
C	-2.764824000	0.261299000	-0.232372000
C	-2.400279000	-1.097183000	-0.518798000
H	-1.382346000	0.674743000	1.349498000
H	-2.472213000	2.066582000	0.829442000
H	-3.556308000	0.712698000	-0.844118000
H	-1.319494000	2.101923000	-1.388613000
H	-1.491788000	-0.761485000	-1.143427000
O	-0.089038000	0.543655000	-1.010012000
C	0.064603000	2.630638000	0.197930000
H	0.513800000	2.173472000	1.090734000
H	0.871148000	2.965244000	-0.469653000
H	-0.534729000	3.500601000	0.497224000
C	-1.858527000	-2.011484000	0.576798000
H	-2.680401000	-2.344198000	1.226234000
H	-1.394978000	-2.893305000	0.118240000
H	-1.095250000	-1.516035000	1.189649000
H	-3.108051000	-1.591106000	-1.195222000
Fe	1.296918000	-0.355068000	-0.036823000
F	0.666580000	-0.235379000	1.680949000

F	2.821240000	0.584559000	-0.196304000
F	1.275721000	-2.056992000	-0.624983000

(*E*)-hex-4-en-2-ol - FeF_3
***E* =** -1873.499425
***G* =** -1873.542065
N_{imag} = 0
***SP* =** -1874.586322

C	-0.325076000	1.894934000	-0.526152000
C	-1.364159000	1.652930000	0.570527000
C	-2.478718000	0.773012000	0.075100000
C	-2.543625000	-0.544651000	0.305053000
H	-0.852613000	1.175903000	1.421269000
H	-1.747082000	2.629806000	0.903633000
H	-3.237472000	1.245030000	-0.563065000
H	-1.770521000	-0.992878000	0.943802000
H	-0.800304000	2.371466000	-1.398527000
H	-0.598947000	0.115407000	-1.368018000
O	0.155664000	0.608798000	-1.003696000
C	0.869500000	2.718260000	-0.096240000
H	1.349985000	2.313359000	0.806503000
H	1.616530000	2.764480000	-0.899835000
H	0.538003000	3.740684000	0.131287000
C	-3.588643000	-1.467971000	-0.237534000
H	-3.123574000	-2.269618000	-0.833337000
H	-4.138481000	-1.962556000	0.578923000
H	-4.311976000	-0.937204000	-0.873496000
Fe	1.288799000	-0.733337000	0.078374000
F	0.559565000	-0.758751000	1.728366000
F	2.922931000	-0.027707000	-0.038795000
F	0.840471000	-2.119831000	-0.956438000

(*Z*)-hex-4-en-2-ol - FeF_3
***E* =** -1873.495771
***G* =** -1873.540165
N_{imag} = 0
***SP* =** -1874.585224

C	-0.861345000	1.450549000	-0.248432000
C	-2.369277000	1.286679000	-0.033691000
C	-2.918147000	0.140578000	-0.839085000
C	-3.103919000	-1.116382000	-0.405470000
H	-2.555896000	1.148723000	1.042197000
H	-2.854645000	2.227927000	-0.330997000
H	-3.127148000	0.350897000	-1.893648000
H	-0.658519000	1.721124000	-1.297877000
H	-0.812548000	-0.528366000	-0.411892000
O	-0.249672000	0.162004000	-0.013261000
C	-0.224710000	2.454324000	0.686534000
H	-0.333048000	2.128424000	1.731103000
H	0.842375000	2.590555000	0.463696000
H	-0.717961000	3.428749000	0.567348000
C	-2.875932000	-1.657058000	0.974787000
H	-3.802155000	-2.105028000	1.367000000
H	-2.124616000	-2.462882000	0.950282000
H	-2.530358000	-0.897032000	1.687694000
H	-3.474264000	-1.844615000	-1.136172000
Fe	1.710168000	-0.370738000	-0.059522000
F	2.303400000	-0.042948000	1.592677000
F	2.350178000	0.697595000	-1.345831000
F	1.324399000	-2.073253000	-0.457172000

Acetaldehyde-$SnCl_4$
***E* =** -2209.195634
***G* =** -2209.237953
N_{imag} = 0
***SP* =** -2209.258416

C	-2.916410000	0.713914000	-0.000673000
H	-3.682901000	1.499237000	0.016512000
O	-1.752913000	1.076909000	-0.006405000
C	-3.406639000	-0.679223000	-0.005890000
H	-2.615394000	-1.416660000	-0.089620000
H	-3.968564000	-0.834069000	0.919068000
H	-4.123887000	-0.787816000	-0.821824000
Cl	1.038330000	2.229681000	0.033761000
Cl	-0.400830000	-0.889275000	1.981713000
Cl	-0.359784000	-0.840082000	-2.019980000
Cl	2.508080000	-0.983671000	0.009983000
Sn	0.379876000	0.018656000	-0.000532000

TS (*E*)-hex-4-en-2-ol: $SnCl_4$
***E* =** -2365.304547
***G* =** -2365.352814
N_{imag} = -298.7498 cm^{-1}
***SP* =** -2366.466498

C	-1.648187000	1.701727000	-0.631393000
C	-2.975852000	1.377914000	0.606163000
C	-3.600057000	0.260600000	0.063174000
C	-3.043206000	-1.073819000	0.164731000
H	-2.355124000	1.216489000	1.493127000
H	-3.537747000	2.315047000	0.580552000
H	-4.431452000	0.418657000	-0.637006000
H	-2.565682000	-1.238765000	1.143113000
H	-2.257888000	1.952824000	-1.514764000
H	-2.151865000	-0.908407000	-0.514038000
O	-0.916968000	0.629376000	-0.833248000
C	-0.989905000	2.932163000	-0.035004000
H	-0.520574000	2.715584000	0.933896000
H	-0.207349000	3.250599000	-0.738860000
H	-1.711853000	3.749182000	0.084718000
C	-3.882367000	-2.247769000	-0.311013000
H	-3.259927000	-3.149206000	-0.384132000
H	-4.701972000	-2.456756000	0.390922000
H	-4.316921000	-2.053720000	-1.302293000
Cl	0.297380000	-2.107005000	-1.144378000
Cl	2.029368000	1.557381000	-1.286279000
Cl	0.044772000	0.214754000	2.164627000
Cl	2.960500000	-1.012637000	0.835100000
Sn	0.910944000	-0.112877000	-0.033667000

TS (*Z*)-hex-4-en-2-ol: $SnCl_4$
***E* =** -2365.303963
***G* =** -2365.350403
N_{imag} = -179.1022 cm^{-1}
***SP* =** -2366.465962

C	-1.853893000	1.555788000	-0.486864000
C	-3.154700000	0.912273000	0.514369000
C	-3.702765000	-0.030012000	-0.366215000
C	-3.155089000	-1.350617000	-0.591460000
H	-2.612323000	0.536007000	1.387941000
H	-3.778874000	1.789743000	0.705367000

H	-4.452408000	0.330201000	-1.081819000
H	-2.381576000	1.920369000	-1.384534000
H	-2.230710000	-1.004186000	-1.152907000
O	-0.991428000	0.595724000	-0.804668000
C	-1.349030000	2.737188000	0.330612000
H	-0.966470000	2.424443000	1.310522000
H	-0.526705000	3.195883000	-0.237122000
H	-2.141116000	3.483648000	0.470187000
C	-2.683819000	-2.179768000	0.598907000
H	-3.540639000	-2.504280000	1.206202000
H	-2.158325000	-3.071692000	0.235011000
H	-1.989027000	-1.623486000	1.240721000
H	-3.762355000	-1.925333000	-1.302219000
Cl	0.451358000	-1.967615000	-1.370124000
Cl	1.844234000	1.861058000	-1.077790000
Cl	0.028612000	0.022199000	2.183967000
Cl	2.995134000	-0.860512000	0.718913000
Sn	0.868780000	-0.042468000	-0.053810000

(*E*)-hex-4-en-2-ol - $SnCl_4$
***E* =** -2365.353154
***G* =** -2365.400876
N_{imag} = 0
***SP* =** -2366.516915

C	-1.872441000	1.847018000	-0.467369000
C	-2.938465000	1.339822000	0.508734000
C	-3.636447000	0.121931000	-0.027671000
C	-3.347732000	-1.131131000	0.345562000
H	-2.453305000	1.114467000	1.470627000
H	-3.656213000	2.157471000	0.677997000
H	-4.382574000	0.285352000	-0.816409000
H	-2.597759000	-1.277045000	1.134864000
H	-2.354562000	2.132650000	-1.417336000
H	-1.537189000	-0.016736000	-1.025353000
O	-0.983091000	0.753341000	-0.802422000
C	-1.071583000	3.020091000	0.052599000
H	-0.583356000	2.779271000	1.007822000
H	-0.306645000	3.324462000	-0.673435000
H	-1.750206000	3.867865000	0.219569000
C	-3.969739000	-2.371133000	-0.213630000
H	-3.196834000	-3.033890000	-0.633931000
H	-4.480387000	-2.943064000	0.577409000
H	-4.699680000	-2.141028000	-1.002956000
Cl	0.074333000	-1.969306000	-1.166868000
Cl	1.988977000	1.610856000	-1.227086000
Cl	0.198108000	0.264565000	2.144630000
Cl	3.030852000	-1.213886000	0.636071000
Sn	1.018266000	-0.140080000	-0.017271000

(*Z*)-hex-4-en-2-ol - $SnCl_4$
***E* =** -2365.351580
***G* =** -2365.399804
N_{imag} = 0
***SP* =** -2366.517047

C	1.978924000	-1.484430000	-0.261391000
C	3.446931000	-1.083616000	-0.079274000
C	3.788434000	0.141701000	-0.882349000
C	3.818690000	1.406004000	-0.432606000
H	3.638282000	-0.930655000	0.993636000
H	4.066859000	-1.933004000	-0.403242000

H	3.973953000	-0.023215000	-1.949410000
H	1.794335000	-1.747197000	-1.316403000
H	1.630364000	0.458206000	-0.341401000
O	1.179176000	-0.318434000	0.041459000
C	1.554320000	-2.624186000	0.637037000
H	1.676599000	-2.352397000	1.695208000
H	0.505969000	-2.900861000	0.459748000
H	2.176099000	-3.504574000	0.422825000
C	3.586767000	1.891609000	0.966887000
H	4.482683000	2.408669000	1.345045000
H	2.768392000	2.628684000	0.981087000
H	3.329073000	1.088411000	1.669495000
H	4.051080000	2.187876000	-1.164721000
Cl	-0.208168000	2.234722000	-0.470829000
Cl	-1.125675000	-1.514351000	-1.720032000
Cl	-1.049122000	-0.656155000	2.202471000
Cl	-3.383422000	0.695318000	-0.116326000
Sn	-1.090858000	0.095459000	-0.012664000

Acetaldehyde-BPh_3
***E* =** -872.419351
***G* =** -872.467982
N_{imag} = 0
***SP* =** -873.684146

C	0.921293000	0.647615000	2.672508000
H	0.864548000	0.450208000	3.757318000
O	0.129851000	0.013663000	1.979052000
B	-0.035071000	-0.061287000	0.326867000
C	1.934021000	1.609021000	2.193571000
H	1.643981000	2.084241000	1.250092000
H	2.145164000	2.349699000	2.974980000
H	2.861062000	1.034440000	2.014643000
C	1.463458000	-0.354976000	-0.221748000
C	2.171710000	0.512869000	-1.065994000
C	2.131865000	-1.520960000	0.193699000
C	3.479897000	0.240639000	-1.472766000
H	1.695892000	1.436095000	-1.408535000
C	3.441389000	-1.800437000	-0.195270000
H	1.610555000	-2.231358000	0.843191000
C	4.122334000	-0.916509000	-1.034428000
H	4.002723000	0.939831000	-2.130329000
H	3.932379000	-2.712743000	0.152935000
H	5.148158000	-1.129724000	-1.344689000
C	-0.722018000	1.328976000	-0.141426000
C	-1.171047000	2.345401000	0.714027000
C	-0.957058000	1.513428000	-1.516948000
C	-1.802988000	3.494751000	0.230051000
H	-1.042688000	2.252665000	1.796368000
C	-1.582215000	2.655448000	-2.012886000
H	-0.644195000	0.736537000	-2.221569000
C	-2.008013000	3.657405000	-1.137742000
H	-2.139014000	4.263784000	0.930265000
H	-1.743204000	2.765266000	-3.088328000
H	-2.499627000	4.554392000	-1.521549000
C	-1.071769000	-1.291659000	0.143766000
C	-2.179792000	-1.438000000	0.995529000
C	-0.959017000	-2.203144000	-0.916956000
C	-3.122066000	-2.448332000	0.809836000
H	-2.309392000	-0.740844000	1.828988000
C	-1.902743000	-3.213592000	-1.120801000

H	-0.109494000	-2.126539000	-1.602415000
C	-2.987263000	-3.342688000	-0.254329000
H	-3.968879000	-2.538148000	1.495452000
H	-1.786213000	-3.907793000	-1.957257000
H	-3.725081000	-4.134413000	-0.406457000

TS (*E*)-hex-4-en-2-ol: BPh$_3$
***E* =** -2516.396720
***G* =** -2516.465098
N_{imag} = -196.7544 cm^{-1}
***SP* =** -2519.643339

C	0.699451000	1.098728000	2.167856000
C	2.236590000	1.770537000	1.714091000
C	3.099595000	0.679158000	1.939728000
C	3.175145000	-0.422542000	1.027695000
H	2.068967000	2.044045000	0.665473000
H	2.376882000	2.620697000	2.387754000
H	3.529746000	0.564005000	2.944085000
H	3.233941000	-0.083351000	-0.017710000
H	0.857520000	0.749549000	3.201935000
H	2.047758000	-0.662792000	1.081030000
O	0.394695000	0.062673000	1.387125000
B	-0.376264000	-0.016473000	0.034699000
C	-0.219907000	2.313012000	2.180893000
H	-0.230925000	2.832827000	1.217884000
H	-1.238782000	1.968325000	2.401477000
H	0.097181000	3.012209000	2.965795000
C	4.042218000	-1.621424000	1.355836000
H	3.766789000	-2.469879000	0.715165000
H	5.106005000	-1.400447000	1.188647000
H	3.913113000	-1.931438000	2.402788000
C	0.201128000	1.101278000	-1.023436000
C	-0.389504000	2.362177000	-1.216186000
C	1.372643000	0.854262000	-1.762700000
C	0.165044000	3.329066000	-2.057772000
H	-1.320835000	2.600145000	-0.694200000
C	1.946777000	1.809886000	-2.602958000
H	1.845280000	-0.129478000	-1.691375000
C	1.345935000	3.060381000	-2.749564000
H	-0.329043000	4.297170000	-2.175365000
H	2.862739000	1.574742000	-3.151642000
H	1.785957000	3.813348000	-3.408085000
C	-1.977578000	0.090829000	0.340313000
C	-2.509414000	-0.233870000	1.599778000
C	-2.906664000	0.385232000	-0.671994000
C	-3.883183000	-0.241832000	1.847421000
H	-1.824503000	-0.491996000	2.413378000
C	-4.283615000	0.378075000	-0.441393000
H	-2.544334000	0.626515000	-1.676071000
C	-4.780864000	0.068844000	0.825150000
H	-4.257017000	-0.495047000	2.843481000
H	-4.974033000	0.616852000	-1.255072000
H	-5.857630000	0.065221000	1.012211000
C	-0.096973000	-1.543672000	-0.484632000
C	-0.349822000	-1.913435000	-1.817332000
C	0.333235000	-2.565968000	0.376426000
C	-0.171439000	-3.219839000	-2.272062000
H	-0.690561000	-1.151590000	-2.525849000
C	0.523269000	-3.878515000	-0.065262000
H	0.519385000	-2.329842000	1.428254000

C	0.273083000	-4.212313000	-1.395663000
H	-0.374966000	-3.465447000	-3.318000000
H	0.864205000	-4.645255000	0.636093000
H	0.420030000	-5.236421000	-1.747971000

TS (*Z*)-hex-4-en-2-ol: BPh_3
***E* =** -2516.395965
***G* =** -2516.465132
N_{imag} = -261.3668 cm^{-1}
***SP* =** -2519.642629

C	-0.531977000	1.152157000	-2.178448000
C	-2.175326000	1.716540000	-2.100712000
C	-2.875521000	0.560918000	-2.505395000
C	-3.131743000	-0.552023000	-1.638326000
H	-2.281389000	2.025463000	-1.055059000
H	-2.202228000	2.545958000	-2.813837000
H	-2.969689000	0.393958000	-3.585554000
H	-0.423397000	0.842981000	-3.233149000
H	-1.998070000	-0.738230000	-1.487267000
O	-0.372846000	0.107887000	-1.368734000
B	0.369245000	-0.089871000	-0.005939000
C	0.285340000	2.418294000	-1.945926000
H	0.000891000	2.948457000	-1.030825000
H	1.343514000	2.132265000	-1.861848000
H	0.168906000	3.090193000	-2.807107000
C	-3.727500000	-0.362481000	-0.248121000
H	-4.824637000	-0.361196000	-0.311115000
H	-3.409630000	-1.182494000	0.410032000
H	-3.410964000	0.581817000	0.209968000
H	-3.548449000	-1.417632000	-2.166500000
C	0.312816000	1.267423000	0.907671000
C	1.446820000	2.039263000	1.207172000
C	-0.912506000	1.757328000	1.392828000
C	1.365755000	3.238634000	1.917952000
H	2.426567000	1.704049000	0.855949000
C	-1.015751000	2.957490000	2.096758000
H	-1.820195000	1.174293000	1.219324000
C	0.129882000	3.710282000	2.359876000
H	2.274126000	3.811568000	2.123366000
H	-1.991391000	3.304475000	2.448023000
H	0.059628000	4.651884000	2.910133000
C	1.895932000	-0.554886000	-0.353717000
C	2.416017000	-0.611988000	-1.654660000
C	2.759094000	-0.954892000	0.683454000
C	3.726492000	-1.027069000	-1.913671000
H	1.778502000	-0.332025000	-2.498364000
C	4.067536000	-1.369493000	0.442770000
H	2.390407000	-0.939366000	1.714789000
C	4.561411000	-1.405291000	-0.864243000
H	4.095927000	-1.056865000	-2.942675000
H	4.708230000	-1.670264000	1.276406000
H	5.586604000	-1.729532000	-1.059886000
C	-0.418240000	-1.354174000	0.674725000
C	-0.748953000	-2.473768000	-0.111685000
C	-0.772306000	-1.419462000	2.030567000
C	-1.433723000	-3.572004000	0.406382000
H	-0.462334000	-2.480352000	-1.168507000
C	-1.448143000	-2.518610000	2.569096000
H	-0.523186000	-0.583473000	2.690253000
C	-1.793597000	-3.596900000	1.756125000

H	-1.683578000	-4.416853000	-0.241494000
H	-1.710184000	-2.529514000	3.630729000
H	-2.328282000	-4.455021000	2.170947000

(*E*)-hex-4-en-2-ol - BPh$_3$
***E* =** -2516.442672
***G* =** -2516.512759
N_{imag} = 0
***SP* =** -2519.689591

C	0.419482000	1.155159000	2.299170000
C	1.793567000	1.830664000	2.196737000
C	2.892763000	0.805880000	2.149165000
C	3.488638000	0.400400000	1.019419000
H	1.808022000	2.458349000	1.291805000
H	1.907936000	2.494780000	3.066862000
H	3.146523000	0.308119000	3.093898000
H	3.223482000	0.906118000	0.081310000
H	0.359035000	0.600559000	3.250683000
H	1.248860000	-0.244461000	1.170805000
O	0.356989000	0.138099000	1.266860000
B	-0.495210000	-0.138380000	-0.166073000
C	-0.745489000	2.115434000	2.211520000
H	-0.665506000	2.776179000	1.339268000
H	-1.699337000	1.573524000	2.155245000
H	-0.752124000	2.731027000	3.122245000
C	4.499979000	-0.694731000	0.903289000
H	4.135444000	-1.471214000	0.211092000
H	5.444536000	-0.312017000	0.485578000
H	4.709485000	-1.162773000	1.875717000
C	-0.672156000	1.303665000	-0.876701000
C	-1.925959000	1.886509000	-1.114086000
C	0.454008000	2.071534000	-1.223078000
C	-2.054618000	3.167509000	-1.655387000
H	-2.831699000	1.333255000	-0.852264000
C	0.343337000	3.354464000	-1.756686000
H	1.453309000	1.657409000	-1.061983000
C	-0.918680000	3.910384000	-1.974245000
H	-3.048222000	3.592029000	-1.820499000
H	1.243355000	3.922792000	-2.005063000
H	-1.015027000	4.915010000	-2.393196000
C	-1.852177000	-0.893751000	0.271055000
C	-2.188557000	-1.213360000	1.594435000
C	-2.745908000	-1.319050000	-0.728952000
C	-3.361285000	-1.904763000	1.911976000
H	-1.515248000	-0.924791000	2.405785000
C	-3.919327000	-2.007661000	-0.426051000
H	-2.515617000	-1.107185000	-1.778301000
C	-4.235079000	-2.302365000	0.902331000
H	-3.590010000	-2.134118000	2.956104000
H	-4.591754000	-2.320094000	-1.229265000
H	-5.154032000	-2.841358000	1.145227000
C	0.554675000	-1.161844000	-0.874992000
C	1.092982000	-0.968782000	-2.156470000
C	0.965858000	-2.317751000	-0.182938000
C	2.010045000	-1.864952000	-2.711518000
H	0.791172000	-0.095059000	-2.739987000
C	1.890291000	-3.212625000	-0.719870000
H	0.543176000	-2.528912000	0.805164000
C	2.420213000	-2.986027000	-1.991149000
H	2.410167000	-1.683789000	-3.712405000

H	2.191739000	-4.095378000	-0.150271000
H	3.142856000	-3.684521000	-2.419917000

(*Z*)-hex-4-en-2-ol - BPh$_3$

***E* =** -2516.439995

***G* =** -2516.510032

N_{imag} = 0

***SP* =** -2519.689591

C	-0.360791000	1.128555000	2.148904000
C	-1.702805000	1.246821000	2.879512000
C	-2.829514000	1.561639000	1.934948000
C	-3.717530000	0.689752000	1.432576000
H	-1.887160000	0.311879000	3.430345000
H	-1.601163000	2.047587000	3.627159000
H	-2.884319000	2.599502000	1.588029000
H	-0.109454000	2.091402000	1.675879000
H	-1.464862000	0.241165000	0.773142000
O	-0.544852000	0.163872000	1.082967000
B	0.413433000	-0.096293000	-0.313485000
C	0.761567000	0.675151000	3.055397000
H	0.517031000	-0.288259000	3.525342000
H	1.704593000	0.556222000	2.507164000
H	0.909788000	1.426074000	3.844328000
C	-3.837032000	-0.776968000	1.716932000
H	-4.837178000	-1.008541000	2.115340000
H	-3.727600000	-1.348810000	0.781058000
H	-3.088141000	-1.145274000	2.430484000
H	-4.451511000	1.084567000	0.720382000
C	1.608273000	-1.057656000	0.187755000
C	2.900393000	-0.968385000	-0.351536000
C	1.380738000	-2.081915000	1.123825000
C	3.919558000	-1.846576000	0.026325000
H	3.120944000	-0.190766000	-1.088312000
C	2.390492000	-2.958171000	1.517751000
H	0.384112000	-2.190457000	1.561239000
C	3.669462000	-2.842985000	0.968244000
H	4.914428000	-1.748144000	-0.415797000
H	2.180678000	-3.737560000	2.255016000
H	4.463891000	-3.528999000	1.272264000
C	0.876833000	1.363971000	-0.846252000
C	0.400961000	1.923356000	-2.044013000
C	1.815854000	2.131612000	-0.130023000
C	0.817037000	3.179289000	-2.493178000
H	-0.307667000	1.360349000	-2.655946000
C	2.232133000	3.391160000	-0.559126000
H	2.251631000	1.726729000	0.787358000
C	1.728934000	3.923486000	-1.747242000
H	0.425721000	3.577409000	-3.432997000
H	2.957942000	3.956570000	0.030757000
H	2.054685000	4.907429000	-2.093634000
C	-0.697816000	-0.853721000	-1.225799000
C	-0.479369000	-2.129911000	-1.768011000
C	-1.949764000	-0.264777000	-1.489835000
C	-1.453151000	-2.786741000	-2.523287000
H	0.477870000	-2.627644000	-1.590976000
C	-2.939086000	-0.916564000	-2.226025000
H	-2.167755000	0.742979000	-1.118487000
C	-2.691676000	-2.186133000	-2.748461000
H	-1.246732000	-3.778838000	-2.932821000
H	-3.901786000	-0.428882000	-2.398524000

H	-3.458552000	-2.702936000	-3.330363000

Acetaldehyde-BMe$_3$
***E* =** -297.969804
***G* =** -298.005761
N_{imag} = 0
***SP* =** -298.473693

C	-1.662119000	-0.856765000	-0.000246000
H	-2.276152000	-1.778973000	-0.000237000
O	-0.451308000	-1.008169000	-0.000380000
B	0.914840000	0.132357000	0.000037000
C	-2.405251000	0.424623000	-0.000019000
H	-1.752927000	1.301927000	-0.001112000
H	-3.068452000	0.433740000	-0.879649000
H	-3.066251000	0.434493000	0.881280000
C	0.744536000	0.960093000	1.375968000
H	0.647393000	0.304221000	2.258460000
H	1.662223000	1.555446000	1.530305000
H	-0.095634000	1.674168000	1.391692000
C	2.139815000	-0.915408000	0.000453000
H	3.093968000	-0.358855000	0.000821000
H	2.149579000	-1.566183000	0.891955000
H	2.150238000	-1.566158000	-0.891059000
C	0.745482000	0.960111000	-1.375992000
H	1.663329000	1.555338000	-1.529838000
H	0.648769000	0.304197000	-2.258501000
H	-0.094588000	1.674288000	-1.392240000

TS (*E*)-hex-4-en-2-ol: BMe$_3$
***E* =** -454.889422
***G* =** -454.928978
N_{imag} = -385.5054 cm^{-1}
***SP* =** -455.672342

C	0.162796000	1.375383000	-0.645798000
C	-1.060055000	1.706469000	0.603796000
C	-2.145318000	0.946170000	0.138521000
C	-2.156441000	-0.480613000	0.235619000
H	-0.551592000	1.333661000	1.498640000
H	-1.191171000	2.791572000	0.573777000
H	-2.855297000	1.428276000	-0.546866000
H	-1.788380000	-0.846722000	1.205734000
H	-0.321166000	1.766074000	-1.557721000
H	-1.173227000	-0.593193000	-0.384715000
O	0.388884000	0.078741000	-0.748383000
B	1.418042000	-0.860548000	0.071421000
C	1.306926000	2.292204000	-0.237184000
H	1.731301000	2.018171000	0.735193000
H	2.095440000	2.202213000	-0.997165000
H	0.968795000	3.336355000	-0.208070000
C	-3.316205000	-1.271075000	-0.336263000
H	-3.035668000	-2.325423000	-0.461688000
H	-4.187484000	-1.232501000	0.333432000
H	-3.619196000	-0.881285000	-1.318775000
C	1.261163000	-0.638099000	1.685753000
H	0.250365000	-0.858965000	2.074523000
H	1.941771000	-1.340243000	2.200123000
H	1.536992000	0.368615000	2.048503000
C	0.943133000	-2.358134000	-0.370985000
H	1.595074000	-3.122694000	0.088284000
H	-0.090202000	-2.600649000	-0.059421000

H	0.996668000	-2.508040000	-1.464741000
C	2.927723000	-0.540605000	-0.463239000
H	3.611370000	-1.345454000	-0.136788000
H	2.982421000	-0.509795000	-1.566991000
H	3.359572000	0.402647000	-0.086639000

TS (*Z*)-hex-4-en-2-ol: BMe$_3$
***E* =** -454.884276
***G* =** -454.923340
N_{imag} = -406.1534 cm^{-1}
***SP* =** -455.667958

C	-0.129078000	1.469476000	-0.472289000
C	-1.602494000	1.378702000	0.428458000
C	-2.384000000	0.587590000	-0.446079000
C	-2.212156000	-0.820863000	-0.554896000
H	-1.300479000	0.916944000	1.375422000
H	-1.927531000	2.418107000	0.531859000
H	-2.883135000	1.108927000	-1.272041000
H	-2.736161000	-1.259949000	-1.411987000
H	-0.439172000	1.972162000	-1.406256000
H	-1.069468000	-0.644338000	-0.853452000
O	0.341007000	0.249286000	-0.729692000
B	1.501238000	-0.618754000	-0.014477000
C	0.733072000	2.427972000	0.338980000
H	0.975477000	2.027561000	1.329489000
H	1.670480000	2.593389000	-0.207573000
H	0.220824000	3.392741000	0.452097000
C	-2.215829000	-1.720791000	0.674049000
H	-1.670840000	-2.651382000	0.470566000
H	-1.749789000	-1.240888000	1.542448000
H	-3.252624000	-1.978550000	0.933504000
C	1.254713000	-0.746977000	1.596881000
H	0.289369000	-1.210035000	1.862187000
H	2.033910000	-1.409806000	2.015780000
H	1.329947000	0.200990000	2.157889000
C	2.939787000	0.070201000	-0.370740000
H	3.752632000	-0.637051000	-0.123976000
H	3.040106000	0.302291000	-1.446935000
H	3.158197000	0.996703000	0.188114000
C	1.345349000	-2.062176000	-0.759902000
H	2.105727000	-2.774873000	-0.393336000
H	0.362059000	-2.537574000	-0.586335000
H	1.480047000	-1.984703000	-1.854315000

(*E*)-hex-4-en-2-ol - BMe$_3$
***E* =** -454.945718
***G* =** -454.986282
N_{imag} = 0
***SP* =** -455.733348

C	0.199541000	1.568253000	-0.510564000
C	-0.975540000	1.617106000	0.471987000
C	-2.157727000	0.858375000	-0.061672000
C	-2.525578000	-0.362126000	0.346244000
H	-0.651252000	1.205369000	1.439399000
H	-1.236638000	2.675473000	0.629272000
H	-2.708834000	1.330617000	-0.886044000
H	-1.965071000	-0.826167000	1.168349000
H	-0.132716000	1.991752000	-1.474243000
H	-0.284055000	-0.277957000	-0.976672000
O	0.542477000	0.195798000	-0.792468000

B	1.551251000	-0.991519000	0.087640000
C	1.425239000	2.325455000	-0.050388000
H	1.792730000	1.951302000	0.914247000
H	2.232408000	2.242980000	-0.790379000
H	1.167420000	3.387608000	0.063821000
C	-3.657492000	-1.162473000	-0.217397000
H	-3.295890000	-2.130569000	-0.599629000
H	-4.402997000	-1.390064000	0.561034000
H	-4.162819000	-0.633260000	-1.038303000
C	1.225807000	-0.839892000	1.661233000
H	0.168603000	-1.025413000	1.915464000
H	1.813897000	-1.605607000	2.198741000
H	1.512857000	0.131115000	2.097702000
C	3.059885000	-0.638124000	-0.351808000
H	3.720166000	-1.463999000	-0.030935000
H	3.175339000	-0.546549000	-1.445342000
H	3.460629000	0.279624000	0.108682000
C	0.949649000	-2.336356000	-0.580576000
H	1.460510000	-3.210319000	-0.140389000
H	-0.129196000	-2.482661000	-0.383747000
H	1.106138000	-2.393375000	-1.671847000

(*Z*)-hex-4-en-2-ol - BMe$_3$
***E* =** -454.944302
***G* =** -454.985823
N_{imag} = 0
***SP* =** -455.731258

C	-0.016010000	1.621524000	-0.375600000
C	-1.358495000	1.369434000	0.315360000
C	-2.298067000	0.641045000	-0.604584000
C	-2.827899000	-0.579284000	-0.443736000
H	-1.183846000	0.819385000	1.251446000
H	-1.783956000	2.350750000	0.582495000
H	-2.536802000	1.174344000	-1.533424000
H	-3.463675000	-0.952588000	-1.255253000
H	-0.204312000	2.182889000	-1.307344000
H	-0.144785000	-0.159712000	-1.204973000
O	0.558312000	0.366276000	-0.793909000
B	1.629526000	-0.825630000	0.011877000
C	0.974187000	2.401335000	0.460243000
H	1.179879000	1.901770000	1.415833000
H	1.920982000	2.535273000	-0.078998000
H	0.553174000	3.394445000	0.670454000
C	-2.685620000	-1.500377000	0.728271000
H	-2.132487000	-2.410371000	0.443932000
H	-2.165879000	-1.037931000	1.577159000
H	-3.677735000	-1.830362000	1.073768000
C	1.084479000	-1.018066000	1.517085000
H	0.044707000	-1.379768000	1.563595000
H	1.705815000	-1.791666000	2.003727000
H	1.160909000	-0.118369000	2.149622000
C	3.103796000	-0.194648000	-0.131790000
H	3.842303000	-0.966995000	0.149270000
H	3.338770000	0.107082000	-1.166826000
H	3.289775000	0.670101000	0.525788000
C	1.329795000	-2.080606000	-0.963066000
H	1.921630000	-2.947104000	-0.620129000
H	0.272488000	-2.406162000	-0.946497000
H	1.611931000	-1.899216000	-2.014851000

Acetaldehyde-Br$_2$
***E* =** -5301.410949
***G* =** -5301.448361
N_{imag} = 0
***SP* =** -5302.277657

C	3.513114000	-0.199263000	0.000072000
H	4.543702000	-0.623804000	0.000249000
O	2.569990000	-0.959511000	0.000012000
C	3.431106000	1.289555000	-0.000027000
H	2.392446000	1.642641000	-0.000273000
H	3.964818000	1.675438000	0.882573000
H	3.965250000	1.675361000	-0.882393000
Br	-2.235929000	0.212704000	0.000025000
Br	0.033316000	-0.305141000	-0.000041000

TS (*E*)-hex-4-en-2-ol - Br$_2$
***E* =** -5458.321416
***G* =** -5458.362718
N_{imag} = -575.7008 cm^{-1}
***SP* =** -5459.465082

C	2.144873000	-1.466099000	-0.397768000
C	2.774845000	-0.758013000	1.160872000
C	3.351026000	0.432117000	0.701210000
C	2.536215000	1.510839000	0.246881000
H	1.835246000	-0.661384000	1.716759000
H	3.453738000	-1.523050000	1.547250000
H	4.409795000	0.417203000	0.410274000
H	1.652651000	1.689838000	0.879238000
H	3.093011000	-1.707890000	-0.918012000
H	2.029762000	0.826969000	-0.587620000
O	1.411654000	-0.598185000	-1.042409000
C	1.461782000	-2.733146000	0.101740000
H	0.617854000	-2.505429000	0.766551000
H	1.073474000	-3.272739000	-0.774755000
H	2.171139000	-3.385836000	0.627813000
C	3.171470000	2.766815000	-0.314988000
H	2.440661000	3.340140000	-0.901096000
H	3.539051000	3.418758000	0.490987000
H	4.018247000	2.524807000	-0.973735000
Br	-3.041789000	0.368749000	0.278110000
Br	-0.794472000	-0.165635000	-0.361883000

TS (*Z*)-hex-4-en-2-ol - Br$_2$
***E* =** -5458.319056
***G* =** -5458.360737
N_{imag} = -645.5966 cm^{-1}
***SP* =** -5459.463744

C	1.972959000	1.273938000	0.200822000
C	3.158296000	0.263607000	1.209727000
C	2.499856000	-0.969938000	1.246074000
C	2.373029000	-1.780690000	0.083141000
H	4.058620000	0.325767000	0.591000000
H	3.226333000	0.823243000	2.146497000
H	1.791337000	-1.151123000	2.062867000
H	1.309107000	1.602178000	1.023657000
H	1.691440000	-0.923933000	-0.418631000
O	1.387608000	0.578862000	-0.737709000
C	2.878516000	2.389754000	-0.295592000
H	3.576764000	2.010251000	-1.055051000
H	2.245228000	3.158314000	-0.763745000

H	3.443861000	2.854850000	0.523780000
C	3.517497000	-1.967518000	-0.901873000
H	4.270588000	-2.659031000	-0.495388000
H	3.137522000	-2.394304000	-1.839683000
H	4.012780000	-1.019075000	-1.147786000
H	1.758438000	-2.677219000	0.227670000
Br	-0.905375000	0.189457000	-0.300369000
Br	-3.209590000	-0.184764000	0.180160000

(*E*)-hex-4-en-2-ol - Br$_2$
***E* =** -5458.384585
***G* =** -5458.427780
N_{imag} = 0
***SP* =** -5459.534234

C	2.339590000	-1.611471000	-0.143915000
C	2.659836000	-0.623117000	0.985705000
C	3.291397000	0.633251000	0.459341000
C	2.647183000	1.794241000	0.292809000
H	1.723203000	-0.388014000	1.517976000
H	3.332995000	-1.120892000	1.701884000
H	4.340820000	0.558772000	0.143716000
H	1.596955000	1.856510000	0.608838000
H	3.284557000	-1.878291000	-0.654975000
H	1.856603000	-0.148055000	-1.333383000
O	1.475956000	-1.007888000	-1.102013000
C	1.675283000	-2.880374000	0.351326000
H	0.746245000	-2.647130000	0.893844000
H	1.427833000	-3.539353000	-0.492920000
H	2.347831000	-3.422843000	1.030511000
C	3.243424000	3.043493000	-0.277643000
H	2.678509000	3.381351000	-1.161069000
H	3.203807000	3.865729000	0.454718000
H	4.292120000	2.894567000	-0.573302000
Br	-3.032638000	0.459919000	0.256103000
Br	-0.903917000	-0.273787000	-0.351117000

(*Z*)-hex-4-en-2-ol - Br$_2$
***E* =** -5458.382060
***G* =** -5458.425247
N_{imag} = 0
***SP* =** -5459.532342

C	1.952186000	1.223860000	0.265811000
C	3.456661000	0.976006000	0.439491000
C	3.733200000	-0.338570000	1.114746000
C	4.076006000	-1.489248000	0.517512000
H	3.937319000	1.030343000	-0.549588000
H	3.869918000	1.798190000	1.044520000
H	3.592877000	-0.353526000	2.201846000
H	1.484526000	1.261304000	1.269020000
H	1.710755000	-0.672851000	-0.107160000
O	1.370499000	0.155661000	-0.476702000
C	1.652146000	2.513774000	-0.470713000
H	2.106104000	2.494227000	-1.472933000
H	0.567409000	2.654319000	-0.581902000
H	2.057315000	3.373032000	0.081992000
C	4.313496000	-1.725009000	-0.944563000
H	5.376297000	-1.949019000	-1.131862000
H	3.743315000	-2.601694000	-1.289331000
H	4.026036000	-0.869248000	-1.569475000
H	4.220325000	-2.363858000	1.162657000

Br	-1.175849000	-0.059138000	-0.175523000
Br	-3.474389000	-0.284045000	0.153298000

Acetaldehyde-Cl$_2$

***E* =** -1073.680417

***G* =** -1073.715713

N_{imag} = 0

***SP* =** -1074.239494

C	-2.698992000	-0.303918000	0.000058000
H	-3.734446000	-0.719487000	0.000453000
O	-1.759230000	-1.065828000	-0.000021000
C	-2.606915000	1.186249000	-0.000088000
H	-1.563971000	1.525847000	-0.002456000
H	-3.138497000	1.578015000	-0.881366000
H	-3.134053000	1.577509000	0.884121000
Cl	0.703236000	-0.303297000	-0.000087000
Cl	2.677955000	0.260400000	0.000063000

TS (*E*)-hex-4-en-2-ol - Cl$_2$

***E* =** -1230.588505

***G* =** -1230.627401

N_{imag} = -661.2017 cm^{-1}

***SP* =** -1231.423871

C	-1.170110000	1.544321000	-0.488491000
C	-1.842688000	0.903470000	1.116586000
C	-2.506524000	-0.244762000	0.676916000
C	-1.775516000	-1.373168000	0.207929000
H	-0.897329000	0.751890000	1.649010000
H	-2.453464000	1.727084000	1.496087000
H	-3.565685000	-0.157379000	0.400991000
H	-0.887057000	-1.610287000	0.813068000
H	-2.109565000	1.882895000	-0.971718000
H	-1.237350000	-0.711514000	-0.643781000
O	-0.549313000	0.602284000	-1.132117000
C	-0.343074000	2.727669000	-0.002506000
H	0.505404000	2.399217000	0.611983000
H	0.054984000	3.245515000	-0.887966000
H	-0.956393000	3.436835000	0.569445000
C	-2.500823000	-2.583548000	-0.344432000
H	-1.825175000	-3.193642000	-0.959463000
H	-2.881413000	-3.222661000	0.466152000
H	-3.352694000	-2.285676000	-0.973383000
Cl	1.555260000	-0.048299000	-0.330015000
Cl	3.434896000	-0.711963000	0.358868000

TS (*Z*)-hex-4-en-2-ol - Cl$_2$

***E* =** -1230.586733

***G* =** -1230.625924

N_{imag} = -780.9060 cm^{-1}

***SP* =** -1231.423068

C	1.056776000	1.306779000	0.114049000
C	2.169295000	0.278230000	1.242511000
C	1.496853000	-0.945249000	1.214145000
C	1.438448000	-1.733757000	0.033060000
H	3.112383000	0.346104000	0.692617000
H	2.161398000	0.840175000	2.180332000
H	0.728178000	-1.125933000	1.974493000
H	0.354551000	1.655656000	0.895644000
H	0.795711000	-0.847380000	-0.502928000
O	0.523013000	0.597844000	-0.834584000

C	2.022960000	2.388722000	-0.339168000
H	2.761129000	1.976185000	-1.041833000
H	1.445879000	3.164733000	-0.864887000
H	2.543442000	2.856837000	0.507856000
C	2.640606000	-1.911431000	-0.881702000
H	3.372074000	-2.603187000	-0.437070000
H	2.321055000	-2.331962000	-1.844783000
H	3.146433000	-0.959510000	-1.090995000
H	0.804549000	-2.624824000	0.116437000
Cl	-3.724276000	-0.232795000	0.194739000
Cl	-1.727519000	0.148711000	-0.324479000

(*E*)-hex-4-en-2-ol - Cl_2
***E* =** -1230.653335
***G* =** -1230.693681
N_{imag} = 0
***SP* =** -1231.495227

C	1.256592000	1.737994000	0.224625000
C	1.668898000	0.817160000	-0.933580000
C	2.449002000	-0.371650000	-0.449666000
C	1.927402000	-1.588095000	-0.250837000
H	0.755798000	0.488544000	-1.456343000
H	2.267860000	1.404409000	-1.647642000
H	3.502945000	-0.198444000	-0.194050000
H	0.870780000	-1.748223000	-0.503781000
H	2.175605000	2.107253000	0.720448000
H	0.964824000	0.209655000	1.388844000
O	0.485554000	1.024450000	1.181460000
C	0.438268000	2.928820000	-0.235414000
H	-0.475720000	2.593268000	-0.748219000
H	0.145971000	3.548572000	0.624187000
H	1.022212000	3.549612000	-0.929543000
C	2.669590000	-2.774667000	0.281160000
H	2.194439000	-3.157972000	1.198320000
H	2.658892000	-3.601701000	-0.446817000
H	3.717273000	-2.530916000	0.510494000
Cl	-1.662897000	-0.003397000	0.325357000
Cl	-3.404387000	-0.899958000	-0.312728000

(*Z*)-hex-4-en-2-ol - Cl_2
***E* =** -1230.651088
***G* =** -1230.692284
N_{imag} = 0
***SP* =** -1231.493479

C	0.848070000	1.171036000	0.226910000
C	2.366403000	1.054868000	0.422603000
C	2.748690000	-0.231896000	1.099830000
C	3.174651000	-1.354629000	0.503193000
H	2.853258000	1.152525000	-0.560324000
H	2.699343000	1.908301000	1.034134000
H	2.617684000	-0.254631000	2.187995000
H	0.364952000	1.158493000	1.223738000
H	0.740260000	-0.734308000	-0.145441000
O	0.369408000	0.070367000	-0.535037000
C	0.448910000	2.439932000	-0.499913000
H	0.920184000	2.474426000	-1.493863000
H	-0.641741000	2.482573000	-0.630063000
H	0.765725000	3.325216000	0.069542000
C	3.416458000	-1.575857000	-0.960463000
H	4.488442000	-1.743205000	-1.154294000

H	2.892042000	-2.481649000	-1.303182000
H	3.078443000	-0.736673000	-1.582319000
H	3.391102000	-2.213933000	1.148954000
Cl	-4.093910000	-0.558024000	0.208495000
Cl	-2.091034000	-0.260846000	-0.165409000

SYNCHRONICITY STUDY

This section summarizes the results obtained from the synchronicity of the carbonyl Alder-ene reaction (computed at PCM(DCM)-ωb97xd/def2-SVP level). The reference system used for the study is the one shown in **Figure S1**.

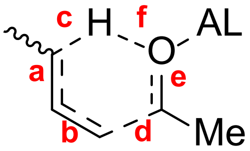

Figure S1. Reference model in the study of synchronicity.

Formation of homoallylic alcohol *E* without catalyst.				
**Ref.**	**Bond**	**WBI reactants**	**WBI TS**	**WBI product**
a	C-C	1.0288	1.2917	1.9123
b	C=C	1.9772	1.4469	1.0295
c	C-H	0.8893	0.5226	0.0043
d	C-C	0.0017	0.4968	0.9811
e	C-O	1.8207	1.3179	0.9079
f	O-H	0.0105	0.2423	0.7531

Formation of homoallylic alcohol Z without catalyst.				
**Ref.**	**Bond**	**WBI reactants**	**WBI TS**	**WBI product**
a	C-C	1.0357	1.3030	1.9184
b	C=C	1.9608	1.4398	1.0234
c	C-H	0.8919	0.5026	0.0036
d	C-C	0.0042	0.4939	0.9798
e	C-O	1.8207	1.3148	0.9060
f	O-H	0.0039	0.2523	0.7547

Formation of homoallylic alcohol *E* using $AlCl_3$ as Lewis acid.				
**Ref.**	**Bond**	**WBI reactants**	**WBI TS**	**WBI product**
a	C-C	1.0310	1.1168	1.9026
b	C=C	1.9441	1.4666	1.0244
c	C-H	0.8935	0.7993	0.0088
d	C-C	0.0264	0.5144	0.9975
e	C-O	1.5341	1.1566	0.7789
f	O-H	0.0017	0.0230	0.6510

Formation of homoallylic alcohol *Z* using $AlCl_3$ as Lewis acid.				
**Ref.**	**Bond**	**WBI reactants**	**WBI TS**	**WBI product**
a	C-C	1.0386	1.1163	1.9080
b	C=C	1.9345	1.4359	1.0202
c	C-H	0.8892	0.8027	0.0087
d	C-C	0.0242	0.5429	0.9989
e	C-O	1.5303	1.1334	0.7767

f	O-H	0.0017	0.0199	0.6521

Formation of homoallylic alcohol *E* using BF_3 as Lewis acid.

Ref.	Bond	WBI reactants	WBI TS	WBI product
a	C-C	1.0301	1.1351	1.9060
b	C=C	1.9532	1.4330	1.0250
c	C-H	0.8978	0.7813	0.0088
d	C-C	0.0204	0.5551	0.9965
e	C-O	1.5634	1.1387	0.7883
f	O-H	0.0011	0.0322	0.6626

Formation of homoallylic alcohol *Z* using BF_3 as Lewis acid.

Ref.	Bond	WBI reactants	WBI TS	WBI product
a	C-C	1.0367	1.1522	1.9046
b	C=C	1.9391	1.3606	1.0214
c	C-H	0.8943	0.7663	0.0113
d	C-C	0.0224	0.6313	0.9957
e	C-O	1.5608	1.0879	0.7876
f	O-H	0.0011	0.0327	0.6526

Formation of homoallylic alcohol *E* using $TiCl_4$ as Lewis acid.

Ref.	Bond	WBI reactants	WBI TS	WBI product
a	C-C	1.0294	1.1073	1.9038
b	C=C	1.9588	1.4926	1.0256
c	C-H	0.8975	0.8062	0.0092
d	C-C	0.0152	0.4809	0.9913
e	C-O	1.6278	1.1762	0.8167
f	O-H	0.0011	0.0182	0.6587

Formation of homoallylic alcohol *Z* using $TiCl_4$ as Lewis acid.

Ref.	Bond	WBI reactants	WBI TS	WBI product
a	C-C	1.0352	1.0980	1.8999
b	C=C	1.9509	1.4797	1.0226
c	C-H	0.8938	0.8204	0.0149
d	C-C	0.0126	0.4847	0.9894
e	C-O	1.6313	1.1511	0.8171
f	O-H	0.0015	0.011	0.6388

Formation of homoallylic alcohol *E* using $FeCl_3$ as Lewis acid.

Ref.	Bond	WBI reactants	WBI TS	WBI product
a	C-C	1.0304	1.1302	1.9069

b	C=C	1.9535	1.4389	1.0248
c	C-H	0.8946	0.7839	0.0070
d	C-C	0.0192	0.5436	0.9945
e	C-O	1.5977	1.1710	0.8073
f	O-H	0.0016	0.0308	0.6780

Formation of homoallylic alcohol *Z* using $FeCl_3$ as Lewis acid.

**Ref.**	**Bond**	**WBI reactants**	**WBI TS**	**WBI product**
a	C-C	1.0379	1.1282	1.9045
b	C=C	1.9409	1.4097	1.0219
c	C-H	0.8891	0.7857	0.0123
d	C-C	0.0190	0.5724	0.9913
e	C-O	1.5952	1.1449	0.8085
f	O-H	0.0023	0.0300	0.6618

Formation of homoallylic alcohol *E* using $FeBr_3$ as Lewis acid.

**Ref.**	**Bond**	**WBI reactants**	**WBI TS**	**WBI product**
a	C-C	1.0304	1.1254	1.9072
b	C=C	1.9538	1.4551	1.0248
c	C-H	0.8946	0.7899	0.0070
d	C-C	0.0186	0.5252	0.9947
e	C-O	1.5954	1.1779	0.8056
f	O-H	0.0021	0.0320	0.6772

Formation of homoallylic alcohol *Z* using $FeBr_3$ as Lewis acid.

**Ref.**	**Bond**	**WBI reactants**	**WBI TS**	**WBI product**
a	C-C	1.0368	1.1250	1.9086
b	C=C	1.9420	1.4130	1.0212
c	C-H	0.8922	0.7944	0.0075
d	C-C	0.0193	0.5662	0.9958
e	C-O	1.5935	1.1387	0.8037
f	O-H	0.0018	0.0236	0.6740

Formation of homoallylic alcohol *E* using $InCl_3$ as Lewis acid.

**Ref.**	**Bond**	**WBI reactants**	**WBI TS**	**WBI product**
a	C-C	1.0285	1.1631	1.9048
b	C=C	1.9745	1.3858	1.0254
c	C-H	0.8935	0.7504	0.0061
d	C-C	0.0008	0.6039	0.9939
e	C-O	1.6265	1.1458	0.8108
f	O-H	0.0014	0.0496	0.6820

Formation of homoallylic alcohol *Z* using $InCl_3$ as Lewis acid.				
**Ref.**	**Bond**	**WBI reactants**	**WBI TS**	**WBI product**
a	C-C	1.0379	1.1895	1.9115
b	C=C	1.9426	1.3127	1.0209
c	C-H	0.8901	0.7257	0.0062
d	C-C	0.0174	0.6811	0.9956
e	C-O	1.6079	1.0915	0.8082
f	O-H	0.0024	0.0561	0.6835

Formation of homoallylic alcohol *E* using FeF_3 as Lewis acid.				
**Ref.**	**Bond**	**WBI reactants**	**WBI TS**	**WBI product**
a	C-C	1.0294	1.1493	1.9106
b	C=C	1.9583	1.3979	1.0246
c	C-H	0.8980	0.7709	0.0049
d	C-C	0.0148	0.5952	0.9934
e	C-O	1.6144	1.1522	0.8143
f	O-H	0.0014	0.0413	0.6909

Formation of homoallylic alcohol *Z* using FeF_3 as Lewis acid.				
**Ref.**	**Bond**	**WBI reactants**	**WBI TS**	**WBI product**
a	C-C	1.0383	1.2231	1.9065
b	C=C	1.9431	1.2616	1.0219
c	C-H	0.8874	0.6954	0.0111
d	C-C	0.0160	0.7406	0.9903
e	C-O	1.6103	1.0546	0.8166
f	O-H	0.0017	0.0676	0.6708

Formation of homoallylic alcohol *E* using $SnCl_4$ as Lewis acid.				
**Ref.**	**Bond**	**WBI reactants**	**WBI TS**	**WBI product**
a	C-C	1.0298	1.1574	1.9046
b	C=C	1.9612	1.4024	1.0259
c	C-H	0.8941	0.7527	0.0088
d	C-C	0.0133	0.5851	0.9920
e	C-O	1.6496	1.1462	0.8201
f	O-H	0.0018	0.0449	0.6737

Formation of homoallylic alcohol *Z* using $SnCl_4$ as Lewis acid.				
**Ref.**	**Bond**	**WBI reactants**	**WBI TS**	**WBI product**
a	C-C	1.0355	1.1728	1.9051
b	C=C	1.9514	1.3285	1.0218
c	C-H	0.8937	0.7410	0.0121
d	C-C	0.0124	0.6641	0.9891
e	C-O	1.6510	1.0865	0.8198

f	O-H	0.0016	0.0417	0.6617

Formation of homoallylic alcohol *E* using $AlMe_2Cl$ as Lewis acid.

Ref.	Bond	WBI reactants	WBI TS	WBI product
a	C-C	1.0293	1.1743	1.9044
b	C=C	1.9564	1.3624	1.0254
c	C-H	0.8972	0.7431	0.0086
d	C-C	0.0174	0.6326	0.9930
e	C-O	1.6004	1.1184	0.8058
f	O-H	0.0014	0.0523	0.6732

Formation of homoallylic alcohol *Z* using $AlMe_2Cl$ as Lewis acid.

Ref.	Bond	WBI reactants	WBI TS	WBI product
a	C-C	1.0363	1.2692	1.9105
b	C=C	1.9450	1.2313	1.0214
c	C-H	0.8928	0.6448	0.0065
d	C-C	0.0171	0.7669	0.9954
e	C-O	1.5972	1.0284	0.8038
f	O-H	0.0020	0.0931	0.6781

Formation of homoallylic alcohol *E* using BPh_3 as Lewis acid.

Ref.	Bond	WBI reactants	WBI TS	WBI product
a	C-C	1.0288	1.2216	1.8939
b	C=C	1.9710	1.2970	1.0267
c	C-H	0.8988	0.6919	0.0164
d	C-C	0.0062	0.6986	0.9897
e	C-O	1.5904	1.0572	0.8023
f	O-H	0.0002	0.0756	0.6265

Formation of homoallylic alcohol *Z* using BPh_3 as Lewis acid.

Ref.	Bond	WBI reactants	WBI TS	WBI product
a	C-C	1.0355	1.2264	1.9013
b	C=C	1.9604	1.2911	1.022
c	C-H	0.8902	0.6755	0.0128
d	C-C	0.0037	0.6982	0.9923
e	C-O	1.5935	1.0586	0.8045
f	O-H	0.0025	0.0781	0.6362

Formation of homoallylic alcohol *E* using BMe_3 as Lewis acid.

Ref.	Bond	WBI reactants	WBI TS	WBI product
a	C-C	1.0290	1.2322	1.9083
b	C=C	1.9640	1.3290	1.0265

c	C-H	0.8983	0.6665	0.0065
d	C-C	0.0114	0.6556	0.9890
e	C-O	1.6500	1.1129	0.8284
f	O-H	0.0011	0.0993	0.6873

Formation of homoallylic alcohol *Z* using BMe_3 as Lewis acid.

Ref.	Bond	WBI reactants	WBI TS	WBI product
a	C-C	1.0352	1.2750	1.9123
b	C=C	1.9534	1.2725	1.0233
c	C-H	0.8985	0.6157	0.0040
d	C-C	0.0109	0.7126	0.9923
e	C-O	1.6516	1.0668	0.8279
f	O-H	0.0003	0.1278	0.6927

Formation of homoallylic alcohol *E* using Br_2 as Lewis acid.

Ref.	Bond	WBI reactants	WBI TS	WBI product
a	C-C	1.0293	1.2546	1.9117
b	C=C	1.9721	1.3602	1.0280
c	C-H	0.8957	0.6244	0.0048
d	C-C	0.0043	0.6146	0.9851
e	C-O	1.7703	1.2059	0.8791
f	O-H	0.0031	0.1467	0.7336

Formation of homoallylic alcohol *Z* using Br_2 as Lewis acid.

Ref.	Bond	WBI reactants	WBI TS	WBI product
a	C-C	1.0363	1.2761	1.9151
b	C=C	1.9588	1.3627	1.0231
c	C-H	0.8923	0.5893	0.0051
d	C-C	0.0047	0.5991	0.9847
e	C-O	1.7701	1.2064	0.8764
f	O-H	0.0026	0.1664	0.7311

Formation of homoallylic alcohol *E* using Cl_2 as Lewis acid.

Ref.	Bond	WBI reactants	WBI TS	WBI product
a	C-C	1.0287	1.2647	1.9119
b	C=C	1.9765	1.3800	1.0278
c	C-H	0.8969	0.6005	0.0050
d	C-C	0.0013	0.5873	0.9833
e	C-O	1.7896	1.2378	0.8860
f	O-H	0.0029	0.1689	0.7373

Formation of homoallylic alcohol *Z* using Cl_2 as Lewis acid.				
**Ref.**	**Bond**	**WBI reactants**	**WBI TS**	**WBI product**
a	C-C	1.0362	1.2863	1.9161
b	C=C	1.9600	1.3822	1.0232
c	C-H	0.8910	0.5634	0.0046
d	C-C	0.0038	0.5719	0.9834
e	C-O	1.7847	1.2359	0.8833
f	O-H	0.0027	0.1893	0.7370
